# Anticancer Effects and Molecular Action of 7-α-Hydroxyfrullanolide in G2/M-Phase Arrest and Apoptosis in Triple Negative Breast Cancer Cells

**DOI:** 10.3390/molecules27020407

**Published:** 2022-01-09

**Authors:** Siriphorn Chimplee, Sittiruk Roytrakul, Suchada Sukrong, Theera Srisawat, Potchanapond Graidist, Kanyanatt Kanokwiroon

**Affiliations:** 1 Department of Biomedical Sciences and Biomedical Engineering, Faculty of Medicine, Prince of Songkla University, Hat Yai, Songkhla 90110, Thailand; siriphorn.chimplee@gmail.com (S.C.); gpotchan@medicine.psu.ac.th (P.G.); 2 Functional Ingredients and Food Innovation Research Group, National Center for Genetic Engineering and Biotechnology (BIOTEC), National Science and Technology Development Agency (NSTDA), Pathum Thani 12120, Thailand; sittiruk@biotec.or.th; 3 Research Unit of DNA Barcoding of Thai Medicinal Plants, Department of Pharmacognosy and Pharmaceutical Botany, Faculty of Pharmaceutical Sciences, Chulalongkorn University, Bangkok 10330, Thailand; suchada.su@chula.ac.th; 4 Faculty of Science and Industrial Technology, Surat Thani Campus, Prince of Songkla University, Surat Thani 84000, Thailand; theera.s@psu.ac.th; 5 Faculty of Innovative Agriculture and Fisheries, Surat Thani Campus, Prince of Songkla University, Surat Thani 84000, Thailand

**Keywords:** 7-α-hydroxyfrullanolide, triple-negative breast cancer, anticancer activity, G2/M-phase arrest, apoptosis, proteomics, sesquiterpene lactone, *Grangea maderaspatana*

## Abstract

Triple negative breast cancer (TNBC) is a breast cancer subtype characterized by the absence of estrogen receptor, progesterone receptor and human epidermal growth factor receptor 2 expression. TNBC cells respond poorly to targeted chemotherapies currently in use and the mortality rate of TNBC remains high. Therefore, it is necessary to identify new chemotherapeutic agents for TNBC. In this study, the anti-cancer effects of 7-α-hydroxyfrullanolide (7HF), derived from *Grangea maderaspatana*, on MCF-7, MDA-MB-231 and MDA-MB-468 breast cancer cells were assessed using MTT assay. The mode of action of 7HF in TNBC cells treated with 6, 12 and 24 µM of 7HF was determined by flow cytometry and propidium iodide (PI) staining for cell cycle analysis and annexin V/fluorescein isothiocyanate + PI staining for detecting apoptosis. The molecular mechanism of action of 7HF in TNBC cells was investigated by evaluating protein expression using proteomic techniques and western blotting. Subsequently, 7HF exhibited the strongest anti-TNBC activity toward MDA-MB-468 cells and a concomitantly weak toxicity toward normal breast cells. The molecular mechanism of action of low-dose 7HF in TNBC cells primarily involved G2/M-phase arrest through upregulation of the expression of Bub3, cyclin B1, phosphorylated Cdk1 (Tyr 15) and p53-independent p21. Contrastingly, the upregulation of PP2A-A subunit expression may have modulated the suppression of various cell survival proteins such as p-Akt (Ser 473), FoxO3a and β-catenin. The concurrent apoptotic effect of 7HF on the treated cells was mediated via both intrinsic and extrinsic modes through the upregulation of Bax and active cleaved caspase-7–9 expression and downregulation of Bcl-2 and full-length caspase-7–9 expression. Notably, the proteomic approach revealed the upregulation of the expression of pivotal protein clusters associated with G1/S-phase arrest, G2/M-phase transition and apoptosis. Thus, 7HF exhibits promising anti-TNBC activity and at a low dose, it modulates signal transduction associated with G2/M-phase arrest and apoptosis.

## 1. Introduction

GLOBOCAN 2020 recently reported that female breast cancer is the most common diagnosis (2.3 million new cases) and the fifth leading cause of cancer mortality (0.68 million cases) worldwide [1,2]. Triple negative breast cancer (TNBC) accounts for 15–20% of total breast cancer cases worldwide [3,4,5,6,7]. The absence of estrogen receptor, progesterone receptor and human epidermal growth factor receptor 2 (HER2) expression in TNBC makes it difficult to treat with chemotherapy [5]. The treatment of patients with TNBC using various standard systematic drugs such as anthracyclines, taxanes and platinum agents has yielded satisfactory results; however, low survival, high rate of relapse, high risk of secondary cancers and strong adverse effects remain serious concerns [8,9,10,11]. Several chemotherapeutic agents derived from terrestrial plants such as taxanes and vinca alkaloids, which contain active compounds, are currently being used for cancer treatment [12,13]. Studies on the identification and development of potential therapeutic drugs from plant sources are continuously being conducted to develop new TNBC treatments.

*Grangea maderaspatana* (L.) Poir., commonly known as Phayaa-mutti, is a medicinal flowering plant belonging to the family Asteraceae (Compositae). This plant is widely found in many tropical countries and often used in various traditional medicine applications, including promotion of menstruation; antispasmodic activity; and relief from stomach ache, coughs and ear ache [14,15]. In Thai ethnopharmacology, this species is referred to as “bitter tonic,” and it is often used for treating flatulence and diarrhea [16]. Methanolic and ethanolic crude extracts and purified flavonoid and sesquiterpenoid extracts of *G. maderaspatana* exhibit antioxidant, anti-microbial and analgesic activities as well as inhibit yeast cell-derived topoisomerase I and II activities [17,18,19,20,21,22]. The crude CHCl_3_ extract of this plant also exhibits strong cytotoxic activity against oral cavity cancer cells (KB cells) [16]. However, there is limited information on the anticancer potential of natural compounds derived from *G. maderaspatana*; consequently, our study is underway to identify these compounds.

Over the last five decades, several natural products derived from sesquiterpene lactones (SLs) have been screened for antitumor activities and their potential activities have been reported in various phases of a previous clinical study [23]. SLs are potential compounds for the development of effective cancer chemotherapies as the anticancer activity of SLs has been reported in more than 60% of studies [24]. Several SLs, including thapsigargin, artemisinin and parthenolide, have exhibited strong activity against certain types of cancers and their associated stem cells [23]. Furthermore, SLs are commonly extracted from Asteraceae plants, which belong to the same family as *G. maderaspatana* [23,24,25,26,27]. The compound 7-α-hydroxyfrullanolide (7HF) is extracted from *G. maderaspatana* and belongs to the eudesmanolide skeleton of SLs. Studies on 7HF have shown it to exert strong biological effects, such as anti-bacterial, anti-inflammatory, and anticancer effects [20,28,29,30,31,32]. Moreover, 7HF isolated from *G. maderaspatana* exerts stronger anti-breast cancer activity on MCF-7 breast cancer cells than on other cancer cell types [20]. However, the anti-breast cancer activity, mode of action and molecular mechanism of action of 7HF in TNBC cells remain unknown. 

In this study, we aimed to estimate the cytotoxicity of 7HF and to this end, we first treated MCF-7, MDA-MB-468 and MDA-MB-231 breast cancer cells and MCF-12A normal breast cells using 7HF and then subjected the cells to MTT assay. Our finding showed that 7HF exhibits anti-TNBC activity and at a low dose, it contributes toward G2/M-phase arrest and apoptosis of cancer cells. 

## 2. Results

### 2.1. Cytotoxic Effects of 7HF on Human Breast Cancer and Normal Cell Lines

**Figure 1:**
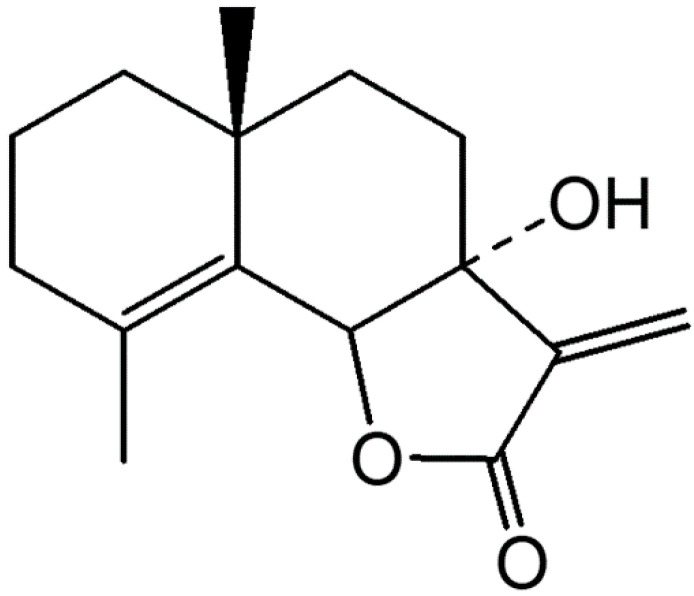
Chemical structure of 7-α-hydroxyfrullanolide extracted from *Grangea maderaspatana*.

The chemical structure of 7HF is shown in Figure 1. Evaluation of the cytotoxicity of 7HF against various breast cancer cell lines at 72 h yielded a series of inhibitory concentrations (ICs): IC_20_, IC_50_ and IC_90_ (Table 1). The inhibitory effects were observed in breast cancer cells rather than in normal cells (Table 1). Findings from the investigation of cytotoxicity at IC_50_ was used for selecting the doses for subsequent experiments. 

The cell survival dose–response curves showed that 7HF inhibited the proliferation of MDA-MB-468 cells more strongly than that of MCF-7 and MDA-MB-231 cells (Supplementary Materials Figure S1). Additionally, the IC_50_ of 7HF in MDA-MB-468 cells (2.97 ± 0.49 µg/mL) was lower than that in MCF-7 (4.05 ± 0.13) and MDA-MB-231 (4.35 ± 0.74) cells (Table 1). The data presented in Table 1 shows that 7HF is less cytotoxic to normal cells (IC_50_ = 12.99 ± 7.42 µg/mL, selective index (SI) > 3) and highly selective toward cancer cells. Therefore, these data indicate that 7HF exhibits strong anti-breast cancer activity and weak cytotoxicity to normal cells.

### 2.2. Mode of Action and Molecular Mechanism of Action of 7HF in TNBC Cells

Application of 7HF significantly reduced the survival of MDA-MB-468 cells in a dose- and time-dependent manner (Supplementary Materials Figure S2). The IC_50_ of 7HF was 3.49 ± 0.22 µg/mL at 24 h which less effective, compared to their survivability at 72 h (2.97 ± 0.49 µg/mL). The IC_50_ (2.97 ± 0.49 µg/mL, 12 µM), half-IC_50_ (1.48 ± 0.24 µg/mL, 6 µM) and double-IC_50_ (5.94 ± 0.98 µg/mL, 24 µM) values of 7HF at 72 h were used as the doses for evaluating the cytotoxic inhibitory activity (in cell cycle arrest and apoptosis analyses) to investigate the mode of action of 7HF at 24 h. The strongest cytotoxicity was observed in 7HF-treated MDA-MB-468 cells, which were selected as the model for subsequent investigations on the mode of action of 7HF in TNBC cells.

#### 2.2.1. 7HF Induces Cell Cycle Arrest and Cellular Apoptosis

**Figure 2:**
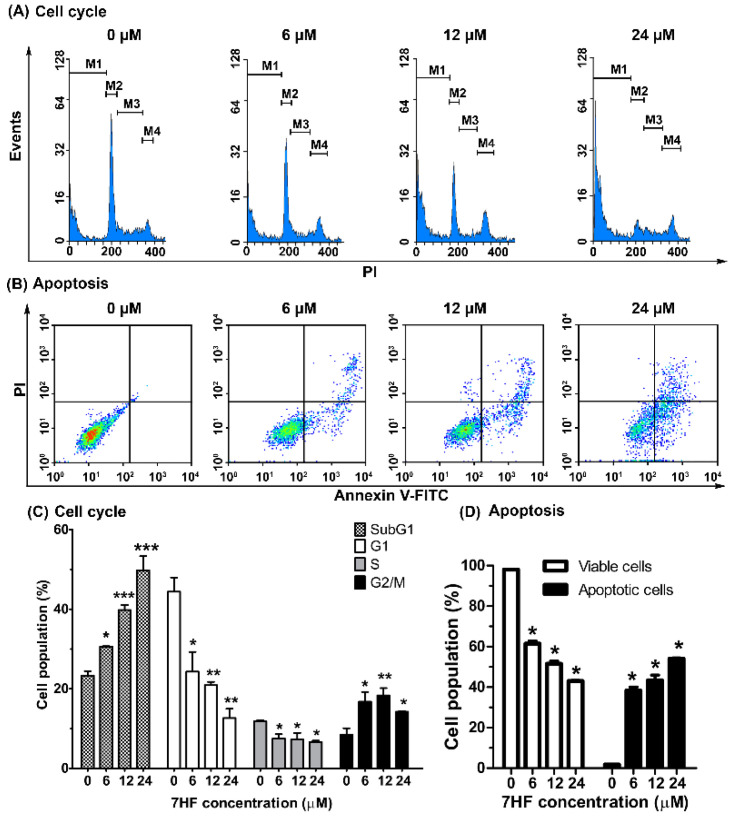
7-α-hydroxyfrullanolide (7HF) induces cell cycle arrest and apoptosis in triple-negative breast cancer cells. The cells were treated with or without 6, 12 and 24 µM 7HF for 24 h, stained with propidium iodide (PI) (for cell cycle analysis) and annexin V-fluorescein isothiocyanate (FITC) and PI (for apoptosis analysis) and analyzed using flow cytometry. (**A**) Representative histograms showing cell cycle distribution. The M1, M2, M3 and M4 annotations in the histograms represent cell populations in the sub-G1, G1, S and G2/M phases, respectively. (**B**) Density plots showing differences in the plasma membrane integrity and permeability based on the number of viable, early apoptotic, late apoptotic, and necrotic cells detected in each 7HF treatment group, as shown in the lower left (V^−^, PI^−^), lower right (V^+^, PI^−^), upper right (V^+^, PI^+^) and upper left (V^−^, PI^+^) quadrants, respectively. (**C**) Data represent the mean ± SD values from two independent experiments of cell cycle analysis. (**D**) The proportion of viable and apoptotic cells was determined in two independent experiments. Differences were analyzed using one-way ANOVA and Tukey’s HSD multiple comparisons (* *p* < 0.05, ** *p* < 0.01, *** *p* < 0.001).

In this study, 7HF treatment significantly expanded the population of cells in the SubG1 phase (from 23.32 to 49.79%) in a concentration-dependent manner (Figure 2A,C). The results were consistent with a density plot, which showed the expansion of the apoptotic cell population to 54% (Figure 2B,D). In terms of the concentration dependence of 7HF cytotoxicity, notably, the population of MDA-MB-468 cells in the G2/M phase expanded upon treatment with 0, 6, 12 and 24 µM of 7HF (increased by 8.49%, 16.76%, 18.34% and 14.23%, respectively) (Figure 2C). Moreover, a significant reduction in both G1 and S-phase cell populations (44.41 to 12.66% and 11.82 to 6.65%, respectively) was observed (Figure 2C). Furthermore, 7HF increased the total apoptotic cell population (1.7 to 54%) and gradually reduced the viable cell population (98.05 to 43.05%) in a dose–dependent manner (Figure 2B,D). These data suggest that 7HF induces G2/M-phase arrest and apoptosis in TNBC cells.

#### 2.2.2. Proteomic Analysis of 7HF-Treated Cells

**Figure 3:**
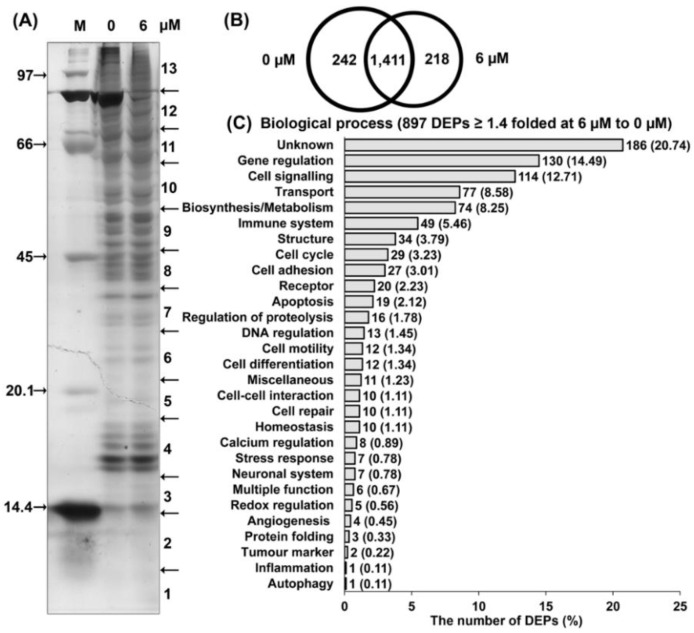
Proteomic analysis of 7-α-hydroxyfrullanolide (7HF)-treated triple-negative breast cancer cells. (**A**) Proteins (10 µg) were separated by 12.5% sodium dodecyl sulfate-polyacrylamide gel electrophoresis and the gel was excised into 13 horizontal slices (1–13). M = Protein marker. (**B**) Venn diagram representing the total number of proteins expressed under individual and shared treatment conditions. (**C**) The significance of the number of differentially expressed proteins (DEPs) under treatment with 6 µM 7HF (≥1.4-fold compared to values in the control) and the biological process classifications.

The 7HF compound was administered at a low dose (6 µM) for 24 h before proteomic analysis to determine the protein expression profiles in TNBC (MDA-MB-468) cells. Sodium dodecyl sulfate (SDS)-polyacrylamide gel electrophoresis (PAGE) revealed the different protein expression profiles between treated and untreated cells (Figure 3A). Proteomic analysis also indicated the presence of numerous proteins. A Venn diagram showing protein expression was used to classify 1411 proteins shared among control cells and cells treated with 6 µM of 7HF (6 µM 7HF-treated cells; Figure 3B). Notably, 242 and 218 proteins were only expressed in control and 6 µM 7HF-treated cells, respectively (Figure 3B).

A total of 897 differentially expressed proteins (DEPs) of 1629 proteins expressed were upregulated or downregulated in 6 µM 7HF-treated cells, with significant differences ≥ 1.4-fold of those in the control (Figure 3C and Supplementary Materials Spreadsheet S1). The biological processes that the DEPs (under 6 µM 7HF treatment) were associated with involved several cell functions (Figure 3C). A total of 162 of 897 DEPs (18.06%) were categorized as cell signaling proteins (114, 12.71%), cell cycle proteins (29, 3.22%) and apoptotic proteins (19, 2.12%) (Figure 3C, Table 2). Moreover, 735 (81.94%) DEPs were associated with the following biological functions: unknown functions (186, 20.74%), gene regulation (130, 14.49%), transport (77, 8.58%), biosynthesis/metabolism (74, 8.25%), immune system (49, 5.46%), structure (34, 3.79%), cell adhesion (27, 3.01%), receptor (20, 2.23%), regulation of proteolysis (16, 1.78%), DNA regulation (13, 1.45%), cell motility (12, 1.34%), cell differentiation (12, 1.34%), miscellaneous (11, 1.23%), cell–cell interaction (10, 1.11%), cell repair (10, 1.11%), homeostasis (10, 1.11%), calcium regulation (8, 0.89%), stress response (7, 0.78%), neuronal system (7, 0.78%), multiple function (6, 0.67%), redox regulation (5, 0.56%), angiogenesis (4, 0.45%), protein folding (3, 0.33%), tumor marker (2, 0.22%), inflammation (1, 0.11%) and autophagy (1, 0.11%) (Figure 3C).

**Figure 4:**
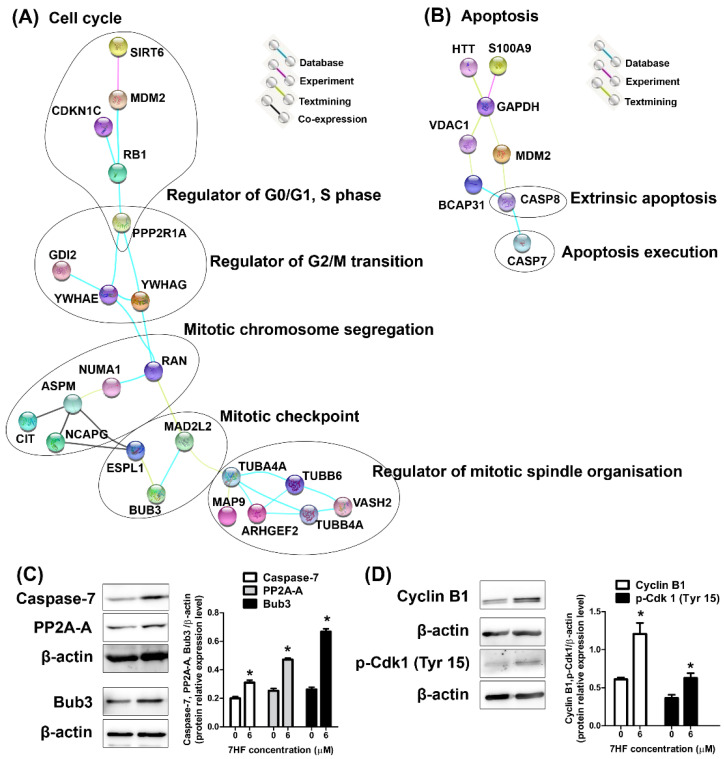
Protein–protein interactions, validated proteins and G2/M-phase-controlled proteins in triple-negative breast cancer cells treated with 6 µM 7-α-hydroxyfrullanolide (7HF) for 24 h. Construction of the (**A**) integrated cell cycle and (**B**) apoptosis signaling networks, respectively. The black drawing indicates the known protein functions. The abbreviations used in STRING represent the protein names shown in Table 2. (**C**) Protein expression was validated using proteomics. (**D**) Cyclin B1 and phosphorylated Cdk1 (p-Cdk1) (Tyr 15) expression after 7HF treatment. Data represent mean ± SD values from three independent experiments. The significance of mean differences was analyzed using one-way ANOVA with Tukey’s HSD multiple comparisons. * *p* < 0.05, compared to the control.

In the present study, protein–protein interactions (PPIs) indicated the molecular signaling networks of cell cycle and apoptosis under treatment with 6 µM of 7HF (Figure 4A,B). The DEPs from STRING analysis associated with cell cycle and cell signaling proteins (as shown in Table 2) are indicated in Figure 4A. The high confidence (≥0.70) of data from the STRING analysis indicated that the proteins were associated with signaling pathways in the G1, S, G2, or M phase. For example, the first serine/threonine-protein phosphatase 2A 65 kDa regulatory subunit A alpha isoform (PPP2R1A) node showed a link with RB1-CDKN1C and RB1-MDM2-SIRT6 signaling. PPP2R1A also interacted with the 14-3-3 adapter proteins YWHAE and YWHAG. The YWHAE-GDI2 and YWHAE/YWHAG-RAN connections were indicated. The second node was a RAN node linked to NUMA1-ASPM-CIT/NCAPG/ESPL1 and MAD2L2-BUB3-ESPL1. Lastly, the TUBA4A node, after MAD2L2 connection, linked to MAP9 and ARHGEF2-TUBB4A-VASH2-tubulin beta chains (TUBB6 and TUBB4A). Moreover, in the apoptosis network, the DEPs related to apoptosis signaling (Table 2) were determined using STRING (Figure 4B). The glyceraldehyde-3-phosphate dehydrogenase (GAPDH) node linked to several molecules such as huntingtin (HTT), S100A9, VDAC1-BCAP31-CASP8-CASP7 and MDM2-CASP8-CASP7. The data networks provided definitive information on 7HF-treated TNBC cells through analyses of cell cycle and apoptotic regulation. 

To validate protein expression based on proteomic data, the proteins PPP2R1A (also known as PP2A-A), caspase-7 and testis mitotic checkpoint BUB3 (Bub3) were selected. Compared with that in the control, the expression of PP2A-A, caspase-7 and Bub3 increased significantly upon treatment with 6 µM 7HF (Figure 4C). The changes in protein expression observed using western blotting were related to the findings of proteomic analysis, thus confirming the reliability of proteomic analysis results. 

#### 2.2.3. Protein Expression in 7HF-Treated Cells

The results of the proteomic and STRING analyses were generally representative of G2/M cell cycle protein expression, which involved the regulation of G2/M-phase transition, mitotic chromosome segregation, mitotic spindle organization and mitotic checkpoint regulation (Figure 4A). To investigate whether 7HF induced G2/M-phase arrest, cyclin B1-phosphorylated Cdk1 (p-Cdk1) (Tyr 15) proteins, which control the G2/M-phase transition, were analyzed. The results showed that the expression of cyclin B1 and p-Cdk1 (Tyr 15) was significantly increased under treatment with 6 µM 7HF for 24 h (Figure 4D). 

**Figure 5:**
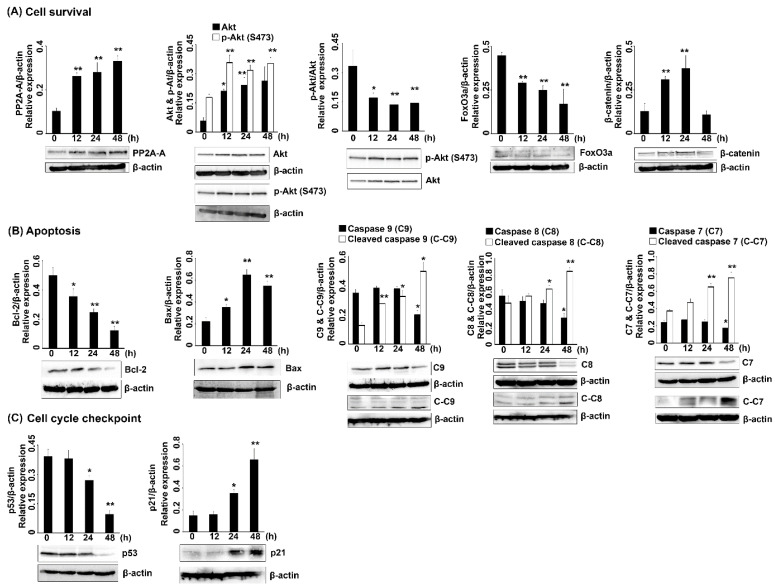
Protein expression in triple-negative breast cancer cells treated with 6 µM 7HF for 0, 12, 24 and 48 h. (**A–C**) The expression of cell survival-related, apoptotic and cell cycle checkpoint proteins and their relative expression, respectively. C-7, C-8 and C-9: caspase-7, 8 and 9; C-C7, C-C8 and C-C9: cleaved caspase-7, 8 and 9. Data represent mean values from three independent experiments. The relative protein expression was statistically analyzed using one-way ANOVA and Tukey’s HSD multiple comparisons test (* *p* < 0.05, ** *p* < 0.01).

Furthermore, the potential inhibitory action of 6 µM 7HF was confirmed by the evaluation of specific signaling pathway-related protein expression at 0, 12, 24 and 48 h. The p53-p21 proteins play an essential role in DNA damage and lead to cell cycle arrest and apoptosis. Here, p53 expression was significantly suppressed in a time-dependent manner; however, the expression of p21 and Bax, which are downstream molecules of p53, increased in a timely manner (Figure 5B,C). The expression of cell survival-regulated proteins is shown in Figure 5A. The expression of PP2A-A, a negative regulator of cell survival, increased gradually in a time-dependent manner. The expression of p-Akt, which positively regulates cell survival, was significantly decreased as the length of treatment with 7HF increased. Meanwhile, the expression of Akt or p-Akt increased gradually. Moreover, the expression of FoxO3a, which downregulates the Akt pathway, was significantly reduced over time and β-catenin expression increased up to 24 h of treatment and then decreased after 48 h of treatment. Furthermore, in the present study, treatment with 6 µM-7HF for 24 h induced significant caspase-7 accumulation (Figure 4C). Several upstream apoptotic molecules associated with the increased expression of the active form of caspase-7 (executioner cleaved caspase-7) were further detected at 0, 12, 24 and 48 h of 7HF treatment. Apoptotic protein expression is shown in Figure 5B. In a time-dependent manner, 7HF gradually reduced the expression of Bcl-2, an anti-apoptotic protein. Bax (pro-apoptotic protein) was increasingly expressed with time dependence. The expression of pro-caspase-7, 8 and 9 proteins diminished with time, especially at 48 h. Meanwhile, the expression of the cleaved isoforms of caspase-7, 8 and 9 increased gradually in a time-dependent manner. Thus, the protein expression findings indicate that 7HF significantly increases cell cycle arrest, apoptosis-related protein expression and decreases cell survival-related protein expression.

## 3. Discussion

In the present study, 7HF exhibited selective cytotoxicity that substantially reduced the survival of TNBC cells. In the evaluation of the range of cytotoxicity of a drug, IC_50_ is the most widely used and informative measure of drug efficacy [37,38]. Additionally, IC_50_ is a convenient indicator of the concentration at which the compound is considered active [39]. According to the standard criterion of the US National Cancer Institute for the preliminary cytotoxic testing of natural products, pure compounds are considered highly strong compounds with an IC_50_ < 4 µg/mL [40]. The anticancer activity of SLs, which depends on the effects exerted by different carbocyclic structures on various types of cancer cells, has been reported in different studies [23,24]. Eudesmanolide, (a 6/6 bicyclic compound), a type of SL, has shown promising anticancer activity [24,25,27,41,42]. As indicated by the findings of the MTT assay at 72 h, 7HF exhibited anti-breast cancer activity, with its IC_50_ ranging from 2.97 to 4.35 µg/mL (11.96 to 17.52 µM). This was similar to the IC_50_ ranges of two novel eudesmanolides, C_17_H_22_O_4_ and C_17_H_25_O_5_, previously reported in five breast cancer cell types [43]. The IC_50_ of C_17_H_22_O_4_ ranged from 3.2 to 11.0 µM (HCC1937 > SK-BR-3 > JIMT-1 > L56Br-C1 > MCF-7) and that of C_17_H_25_O_5_ ranged from 9.3 to 27.0 µM (L56Br-C1 > SK-BR-3 > JIMT-1 > HCC1937 > MCF-7) [43]. Additionally, eight eudesmanolide SLs (IJ-1, 3, 5, 6, 9 and 11 and IH-45 and 49) exerted more potent anti-TNBC activity (MDA-MB-231) than anti-non-TNBC (MCF-7) activity at 72 h [44]. The IC_50_ ranged from 10 to 50 µM [44]. Moreover, frullanolide exhibited anti-breast cancer cell activity on MDA-MB-468, MCF-7 and MDA-MB-231 cells, with IC_50_ values of 34.61, 46.23 and 53.20 µM, respectively [27]. The compound 7HF exhibited strong activity against TNBC cells (MDA-MB-468 cell line) and this was concordant with the cytotoxicity of frullanolide [27]. However, 11.97 µM of 7HF exhibited better potential for reducing viability of MDA-MB-468 cells than 34.61 µM of frullanolide. Therefore, in this study, 7HF-treated MDA-MB-468 cells were selected as model cells for investigating the mode of action of 7HF in TNBC as the cells showed the highest anticancer activity. Additionally, MDA-MB-468 cells are one of the most important cell types that express high levels of EGFR on their membrane [45,46,47]. EGFR is a target for novel anticancer agents and almost 50% of patients with TNBC show EGFR overexpression [48,49]. Furthermore, several studies have attempted to screen 7HF activity in different types of human cancers [16,20,31,32]. Nonetheless, despite their importance, the anti-breast cancer activity and proposed molecular mechanism of action of 7HF in TNBC cells remain to be elucidated. To the best of our knowledge, this is the first study to report the activity–action network of 7HF-treated TNBC cells. 

Natural compounds with IC_50_ > 10 µg/mL and SI > 3 have been considered to exert weak cytotoxicity against normal cells and selective cytotoxicity against cancer cells [34,35,36]. This is in accordance with our finding that 7HF is highly selective for breast cancer cells (IC_50_ ranging from 2.97 to 4.35 µg/mL). The low selective cytotoxicity or low harmful potential of 7HF is applicable to normal cells (IC_50_ = 12.99 µg/mL, SI = 2.99–4.37). 

The first mode of action of low-dose 7HF in TNBC cells is primarily associated with the signal transduction pathway in G2/M-phase arrest. Cells treated with 7HF showed high accumulation at the G2/M phase (based on the measurement of cellular DNA content by flow cytometry) and concomitantly expressed Bub3, cyclin B1, p-Cdk1 (Tyr 15) and p21 proteins at high levels. In general, in the G2/M phase in human cells, cyclin B1 expression is temporally restricted to the G2 phase and early mitosis transition [50]. In the late G2 phase, cyclin B1 forms a complex with Cdk1 (cyclin B1-Cdk1 activity is driven by the p-Cdk1 (Thr 161) subunit) to promote cell division through mitosis [51,52]. Meanwhile, in a feedback loop of the cyclin B1-Cdk1 network, unphosphorylated Cdk1 is inactivated by Wee1 kinase at Tyr 15 in the nucleus, which helps maintain cyclin B1 levels [53,54]. The increased levels of p-Cdk1 (Tyr 15) have been shown to be associated with G2/M-phase arrest, following DNA damage, in different cell types [53,54]. Moreover, higher levels of cyclin B1-Cdk1 are more important for mitotic spindle assembly than for mitosis initiation [55]. Lindqvist et al. (2007) suggested that sustained high cyclin B1-Cdk1 activity is critical for retaining cells in the mitotic state for as long as necessary to ensure the attachment of all chromosomes to the mitotic spindle [55]. In case unaligned chromosomes are present in cells that require mitotic spindle checkpoint activation, the core effector of the mitotic checkpoint complex (MCC), which is composed of Bub3, BUBR1, MAD2 and CDC20, is activated [56,57]. The MCC is an effector that mediates signal transduction only for inhibiting the activity of anaphase-promoting complex/cyclosome (APC/C) (an E3 ubiquitin ligase) in mitosis [56,58,59]. APC/C^cdc20^ targets securin and cyclin B1 degradation and induces anaphase onset [56,57,60]. Separase is maintained in an inactive state by securin and Cdk1-cyclin B and sister chromatids are held together by cohesins [60]. Cyclin B1 and securin are accumulated in response to APC/C^cdc20^ inhibition [60]. Cells treated with 7HF showed increased expression of separase (*ESPL1*); however, we suggest that securin expression should be determined to confirm the occurrence of mitotic arrest. Cyclin B1-Cdk1 activity also facilitates the release of MAD1 (an MCC protein) from the nuclear pore to ensure a robust spindle checkpoint and cyclin B1-MAD1 binding is necessary to induce cyclin B1 accumulation at unattached kinetochores [61]. In the present study, Bub3 protein expression was upregulated, as indicated by the results of both proteomic analysis and western blotting. Bub3 is ubiquitously expressed throughout the cell cycle in both interphase and mitotic cells and Bub3 and its partner, BubR1, form free in the cell cycle phases [56,58]. Nevertheless, Bub3 is an evolutionarily conserved protein of the MCC [56,58,62]. BUB3:BUBR1 forms a mitosis-dependent complex under high CDC20 and MAD2 expression [56,59]. In accordance with our findings, the expression of the mitotic assembly checkpoint protein, MAD2B (*MAD2L2*), a subtype of the MAD2 unit of the MCC, was also upregulated, as indicated by proteomic analysis. Bub3 was thus selected for guiding action related to 7HF-induced G2/M-phase arrest, which led to the transmission of the MCC signal through the upregulation of cyclin B1 expression. Furthermore, a Bub3 spindle checkpoint protein has been shown to act as a tumor suppressor after apoptosis inhibition and mediate cell cycle arrest after paclitaxel treatment [63,64]. Kinetochores unattached to the mitotic spindle of MCF-7 breast cancer cells treated with nocodazole, a prototypic microtubule inhibitor, are bound by the spindle checkpoint protein, MAD2, which induces prometaphase arrest. The feedback upregulation of cyclin B1 and Cdk1 protein expression strongly control the arrest [65]. Many studies have reported natural product or SLs compounds were related to the G2/M arresting pathway through dependent cyclin B1 and/or p-Cdk1 (Tyr 15) increases. Meanwhile, the effects of natural compounds on Bub3 activity remain undetermined. Harmaline, an alkaloid, induced G2/M-phase arrest by upregulating cyclin B1 and p-Cdk1 expression in SGC-7901 gastric cancer cells [66]. Gallic acid induced early Tyr 15-mediated Cdk1 phosphorylation [67]. Several studies have reported the mechanisms of action of SLs. For example, santamarine (a eudesmanolide) induced G2/M-phase arrest in a murine leukemia model [68]. Costunolide, a eudesmanolide skeleton, induced mitotic arrest, but not progression to the G2 phase, by upregulating the expression of p-Cdk1 (Tyr 15) and cyclin B1 in HA22T/VGH hepatoma cells [69]. Furthermore, pseudoguaianolide (a 5/7 bicyclic compound) SLs of 6-O-angeloylplenolin, coronopilin, pulchelloid A and hymenoratin caused G2/M-phase arrest by activating cyclin B1-Cdk1/p-Cdk1 in multiple myeloma, leukemia and colon cancer cells, respectively [69,70,71,72,73]. 

Cyclin-dependent kinase inhibitor 1 or CDK-interacting protein 1, also known as p21 (WAF1/Cip1), is a cyclin B1-Cdk1 complex inhibitor [74]. In comparison with SLs, such as costunolide [75] and dehydrocostus lactone [76], 7HF upregulated p21 expression independent of cyclin B1-p-Cdk1 inhibition. Similar to calein C (based on a eudesmanolide skeleton), 7HF induced p21 accumulation by inducing stable cyclin B1 expression and suppressing the expression of polo-like kinase 1 and aurora kinase B, which inhibited Cdk1 mitotic progression and induced mitotic arrest in non-TNBC cells (MCF-7 cells) [77]. 

The second mode of action of 7HF might be delineated by the upregulation of *PPP2R1A* (also known as PP2A-A or scaffold A subunit) expression. Upregulation of the expression of the PP2A-A subunit has been associated with the aberrance of several protein kinases in cell cycle regulation [78]. The active PP2A phosphatase serves as a scaffolding subunit to coordinate the assembly of the catalytic (C) subunit and a variable regulatory (B) subunit that mediates subcellular localization and substrate specificity [79]. PP2A also plays a pivotal role as a tumor suppressor and anticancer therapeutic agent in many cancer cells [79]. In this study, we found that the PP2A-A subunit might indirectly enhance PP2A phosphatase activity mediated through the PI3K-Akt survival transduction pathway [80,81,82]. PP2A-A may dephosphorylate Akt at Ser 473, an indicator of Akt activity, thereby reducing p-Akt levels. The downregulation of p-Akt promotes the expression of phosphorylated FoxO proteins including FoxO3a [83]. In contrast to our results, 7HF-treated cells showed FoxO3a suppression after the downregulation of p-Akt. However, Jin et al. (2004) reported that in patients with breast cancer, FoxO3 expression is associated with lymph node metastasis and poor prognosis [84]. The phosphorylation at Ser 473 in Akt is also correlated with the poor prognosis of patients with breast cancer and melanoma [85,86]. In the case of Akt–FoxO signaling, the downregulation of p-Akt and FoxO3 expression could be a consequence of 7HF treatment, which could reduce the aggressiveness of breast cancer. Moreover, natural compounds have been shown to suppress PI3K–Akt signaling in numerous cancer cells [87]. In a previous study on the MCF-7 cell line, tehranolide, an SL, inhibited cell proliferation by downregulating p-Akt and p-PI3K expression [88]. In the oxidative stress response, phosphorylated FoxO forms a complex with β-catenin and induces cell cycle arrest and apoptosis [89]. In the present study, reduction in FoxO protein levels suggested the inhibition of β-catenin complex formation at 48 h. Concomitantly, the downregulation of β-catenin expression may be negatively regulated by AKT-inhibited PP2A-A scaffold expression through Wingless signaling [79,90,91,92]. Potent SLs, such as β-elemene and shizukaol D, have been reported to reduce cervical and liver cancer cell proliferation by suppressing β-catenin modulation [92,93].

The last mode of action of low-dose 7HF could be mapped to the induction of apoptosis-related signaling. This is because 7HF induced the accumulation of apoptotic cells (based on plasma membrane integrity and permeability assessed using flow cytometry) and modulated the expression of proteins involved in intrinsic and extrinsic apoptotic pathways; these findings are consistent with those reported by Fulda and Debatin, (2006), Elmore (2007) and Burz et al. (2009) [94,95,96]. In the present study, the expression of anti-apoptosis (Bcl-2) proteins upstream of intrinsic apoptotic proteins was gradually decreased in 7HF-treated cells. Moreover, the expression of pro-apoptosis proteins (e.g., Bax) gradually increased. The initiator intrinsic caspase-9 gradually converted into active cleaved caspase-9. In a distinct pathway, initiator caspase-8 increasingly converted to active cleaved caspase-8 (as assayed using proteomics and western blotting), which indicated extrinsic apoptotic activity. The effector caspase-3/7, which converted to cleaved caspase-3/7 after initiator caspase signals occurs in both intrinsic-dependent mitochondrial and extrinsic-dependent death receptor apoptosis pathways [97]. In 7HF-treated cells, the upregulation of caspase-7 expression was observed using proteomics and its gradual downregulation in a time-dependent manner was observed using western blotting. Subsequently, the levels of cleaved caspase-7 increased concomitantly with displacement by 7HF. Natural compounds such as matrine (alkaloid), quercetin (flavonoid), curcumin (curcuminoid) and triptolide (diterpenoid) have been previously applied to cancer therapy based on their ability to target apoptotic pathways [98]. The mechanisms of action of the compounds are similar to those of 7HF, which induced apoptosis through both intrinsic (modulation of Bcl-2 expression and upregulation of caspase-9 expression) and extrinsic (upregulation of caspase-8 and death receptor expression) pathways [98]. Notably, alantolactone, a eudesmanolide SL, also plays a critical role in both types of apoptosis by regulating Bax expression; activating caspase-9, 8 and 3 and cytochrome c; and downregulating Bcl-2 expression [24,98]. Colon cancer cells treated with 7HF have also shown concurrent upregulation of caspase-8, 9 and 3 expression (in intrinsic and extrinsic pathways) [32]. Furthermore, apoptosis induction is mediated through p53-dependent activation in DNA damage-presenting cells and leads to cell cycle arrest by the downstream molecule p21 [99,100]. The results of the present study confirmed that p53-independent apoptosis induction and p53-independent p21 activation lead to cell cycle arrest at the G2/M phase. The reduction of p53 expression under 7HF treatment may induce p53 mutation in MDA-MB-468 cells and p53 degradation via MDM-2 regulation [101,102]. The upregulation of MDM-2 expression was also observed in 7HF-treated cells using proteomics. Ozaki and Nakagawara (2011) reported that p53-mutated cells can maintain p53 expression with a half-life of approximately 2 to 12 h and followed degradation by MDM-2 [102], which corresponded to a considerable reduction in p53 expression after 24 to 48 h of 7HF treatment in MDA-MB-468 cells. However, Pandey et al. (2019) suggested that the treatment of colorectal cancer cells with 7HF did not affect the efficiency of apoptosis via p53-dependent (p53^+/+^) or p53-independent (p53^−/−^) mechanisms in vivo [32]. 

Notably, our findings from proteomic identification and STRING analysis revealed the PPIs and their biological functions were related to cell cycle and apoptosis. The details of the protein clusters involved are provided for a better understanding of the effects of 7HF treatment on TNBC cells. The first node is the PPP2R1A-RB1-CDKN1C/MDM2-SIRT6 node associated with G1/S-phase arrest. For example, PP2A-A and its core dimers (B and C subunits) induce the dephosphorylation of Rb and suppression of p-Rb-E2F, leading to G1/S-phase arrest [103,104]. CDKN1C/p57^Kip1^ inhibitor also mediates defective Rb-E2F and Cdk2-cyclin E formation and G1-phase arrest [105]. RB1 and SIRT6 expression may be maintained by MDM-2 degradation. NAD-dependent protein deacetylase sirtuin-6 (SIRT6) induces the deacetylation of telomeric histone H3 lysine K56 during the S phase, which is required for proper telomere metabolism and function [106,107]. The second mode is the PPP2R1A-YWHAE/YWHAG (14-3-3 family)-GDI2/RAN node associated with the regulator of G2/M-phase transition. PP2A associated with 14-3-3 interaction act to maintain Cdc25 members for CDK1 regulation in G2/M [108,109]. Proteins of the 14-3-3 family also act as regulator adapters in several signal transduction pathways, including GDP- and GTP-bound switching in cell cycle; therefore, the activities of Rab GDP dissociation inhibitor beta (GDI2) and GTP-binding nuclear protein Ran (RAN) may be controlled by 14-3-3 proteins [110]. The third mode is the RAN-nuclear mitotic apparatus protein 1 (NUMA1)-abnormal spindle-like microcephaly-associated protein (ASPM)-Citron rho-interacting kinase (CIT)/chromosome-associated protein G (NCAPG)/ESPL1 (Separin) node. Notably, all detectable proteins were associated with regulatory functions in mitosis, such as mitotic spindle assembly, mitotic spindle pole organization, mitotic chromosome segregation and cytokinesis [111,112,113,114,115,116,117]. The last node is the MAD2L2-TUBA4A/MAP9-TUBB6/TUBB4A-ARHGEF2-VASH2 node, which is related to microtubule dynamics or organization. MAD2L2 is a MCC component associated with the mitotic spindle checkpoint of microtubules formed between tubulinα/β dimers (TUBA4A; tubulin alpha-4A and TUBB6/TUBB4A; tubulin beta-6/4A chain) [56,57]. Rho guanine nucleotide exchange factor 2 (ARHGEF2) is responsible for GDP–GTP switching for the regulation of microtubule dynamics [118,119,120]. Microtubule dynamics organization is also regulated by microtubule-associated proteins (e.g., MAP9) [121] and vasohibin-2 removes tyrosine from microtubules to control mitotic spindle length and positioning [122]. The molecular mechanism of action associated with apoptosis in 7HF-treated cells, preliminarily indicated by the results of proteomics and STRING analyses in this study, should be investigated further; some of the molecules shown to be involved in this process are mentioned as follows: GAPDH forms a central node in HTT/S100A9/VDAC1/MDM2 interactions. The functions of GAPDH and their partner interactions, except GAPDH-voltage-dependent anion channel 1 (VDAC1), remain unknown [123]. GAPDH interacts with VDAC1 and may participate in intrinsic apoptosis-associated mitochondrial membrane permeabilization and permeability transition pore complex formation [123]. Moreover, S100A9 has been shown to induce mitochondria-dependent apoptosis via the translocation of BNIP3, a pro-apoptotic Bcl-2 family member [124]. Further, HTT acts as a substrate of the executioner caspase cleaved caspase-3 [125]. B-cell receptor-associated protein 31 (BCAP31), a chaperone protein, may mediate extrinsic apoptosis through caspase-8 interaction [126]. Furthermore, the cytotoxicity assessment in vivo will be performed for further study.

## 4. Materials and Methods

### 4.1. Plant Material and Isolation

The identity of *G*. *maderaspatana* (L.) Poir. was confirmed according to a method reported by Ruangrungsri et al. (1989) [16]. A voucher specimen (No. 5182) was deposited at the Museum of Natural Medicine, Chulalongkorn University, Bangkok, Thailand. An extract was prepared from the powdered plant material (1500 g) by maceration with dichloromethane (CH_2_Cl_2_). The extract was evaporated under reduced pressure at 55 °C. The CH_2_Cl_2_ extract (4.25 g) was separated by vacuum liquid column chromatography using silica gel (No. 7734, 200 g). Elution was performed using a polarity gradient with mixtures of CH_2_Cl_2_ and acetone (10:0 to 0:10). The mixtures were sequentially fractionated using a Sephadex LH20 column with a mixture of CH_2_Cl_2_ and MeOH (in a 1:1 ratio) and by column chromatography (silica gel No. 9385) with gradient mixtures of Hexane-EtOAc (10:0 to 0:10). The compound was obtained as a yellow oil (225.5 mg, Rf. 0.74, silica gel, CH_2_Cl_2_-Acetone 9:1). The structure of 7HF was determined using ^1^H, ^13^C NMR spectroscopy and mass spectrometry (MS). The physical and spectral data were compared with those reported in a previous study [16]. The compound was identified as 7HF, as shown in Figure 1, with the chemical formula C_15_H_20_O_3_ (peak of parent compound at m/z 248). The purity of 7HF was more than 90%. The compound was stored at −20 °C prior to testing on cancer cells. 

### 4.2. Cell Culture

TNBC cells (MDA-MB-468 and MDA-MB-231), non-TNBC cells (MCF-7) and normal breast cells (MCF-12A) were purchased from the American Type Culture Collection (ATCC, Manassas, VA, USA). The cells were previously cultured in growth media under specific conditions, as reported by Chimplee et al. (2019) [27].

### 4.3. Cell Viability Assay

The anticancer activity of 7HF on breast cancer and normal cells was assessed in the MTT assay. After seeding (2 × 10^4^ cells/well), the cells were treated with 1.25–20 µg/mL 7HF for 72 h. DMSO (0.02%) and doxorubicin (0.1–10 µM) were used for treating the negative and positive controls, respectively. After 7HF treatment for 72 h, MTT assay was performed to measure cell viability, as described previously [27]. The IC_20_, IC_50_ and IC_90_ values were calculated from a fitted semi-log response curve plotted using the cell viability (%) and concentration. Data are presented as the mean ± SD from three independent replicates. SI was calculated using the following formula: 

IC_50_ of normal cells/IC_50_ of cancer cells.

### 4.4. Flow Cytometry for Cell Cycle Analysis and Apoptotic Cell Detection

MDA-MB-468 cells were seeded (4 × 10^5^ cells/well) and synchronized by overnight treatment with 0.5% fetal bovine serum for cell cycle analysis. The cancer cells were then treated with 7HF at half-IC_50_ (6 µM), IC_50_ (12 µM) and double-IC_50_ (24 µM) for 24 h. Prior to the evaluation of cell cycle distribution and cell death using flow cytometry (FACSCalibur, Becton Dickinson Biosciences, San Jose, CA, USA), the cancer cells were prepared as described by Chimplee et al. (2019) [27]. Briefly, the cancer cells for cell cycle analysis were fixed in cold 70% EtOH (for 4 h), following which DNA was removed by treating the cells with 100 µg/mL ribonuclease A. Next, the cells were stained with 50 µg/mL PI. Cell death analysis was performed using annexin V-FITC and PI double-staining, according to the manufacturer’s instructions (BD Biosciences, San Jose, CA, USA). In each experiment, data for 5 × 10^3^ cells were acquired using the CellQuest program (Becton Dickinson Biosciences). WinMDI program version 2.9 (Scripps Institute, San Diego, CA, USA) was used to generate histograms for analyzing the cell cycle and construct density plots for analyzing the proportion of apoptotic cells. Two independent experiments were performed in triplicate.

### 4.5. Proteomic Analysis

#### 4.5.1. Sample Preparation and One-Dimensional PAGE

After treatment with 6 µM 7HF for 24 h, MDA-MB-468 cells were lysed using 0.5% SDS. Protein quantification was performed using the Pierce Micro BCA Protein Assay Kit (Thermo Fisher Scientific, Waltham, MA, USA) per manufacturer’s instructions. In brief, absorbance was measured at 562 nm and the protein concentration was calculated from a standard curve based on the optical density at 562 nm and BSA concentrations. Equal quantities of proteins (10 µg) were loaded on a 12.5% SDS-polyacrylamide gel and a low-range marker (Amersham Biosciences, Little Chalfont, Bu, UK) was used as the standard protein. Next, the samples were separated in Tris-glycine buffer (25 mM Tris-HCl, pH 8.3, 192 mM glycine and 0.1% SDS). The gel was double-stained with Coomassie Brilliant blue R-250 and a silver dye and destained using a destaining solution consisting of 16.5% ethanol and 5% glacial acetic acid. The gel was then observed using a GS-710 imaging scanner (Bio-Rad Laboratories, Inc., Hercules, CA, USA). The parts of the gel with the protein samples were excised to produce 13 slices with low to high molecular weights of proteins (Figure 3A). The gel slices up to a size of 1 mm^3^ were excised and transferred to each well of a low-binding 96-well plate. The experiments were performed in triplicate.

#### 4.5.2. In-Gel Digestion

The excised, stained gel pieces were destained using 25 mM ammonium bicarbonate (NH_4_HCO_3_) in 50% MeOH and further dehydrated with 100% acetonitrile (ACN). The dried gel pieces were reduced and alkylated by treatment with 10 mM dithiothreitol and 100 mM iodoacetamide in 10 mM NH_4_HCO_3_ at 56 °C and room temperature for 1 h in the dark. After washing with 100% ACN, the dried alkylated gels were subjected to reswelling by treatment with trypsin (10 ng/µL in NH_4_HCO_3_) and then incubated overnight at 37 °C. To collect the peptide solution, 20 µL of 20% ACN was added to the gel and 50% ACN in formic acid (FA, 0.1%) was used to extract the peptides (3 × 10 min with repeated pipetting). Subsequently, the supernatant was dried overnight at 40 °C and stored at −20 °C prior to MS injection (Waters Corp, Milford, MA, USA).

#### 4.5.3. LC-MS/MS, Protein Identification and Bioinformatics Analysis

The extracted peptides were resuspended in 20 µL of FA. After centrifugation, 12 µL of the peptide solution was injected into the LC-MS/MS instrument (ESI-QUAD-TOF). The peptide sequences were analyzed using the DeCyder MS 2.0 Differential Analysis Software (GE Healthcare Bio-Science AB, Uppsala, Sweden) and the Mascot search program, with identification using the NCBInr database (the workflow is shown in Supplementary Materials Figure S3). For additional parameters of the mascot MS/MS ion search, *Homo sapiens* (for taxonomy) and trypsin digestion were selected. Up to three missed cleavages were allowed and the fixed and variable modifications were set to carbamidomethyl (C) and oxidation (M), respectively. The respective tolerance values of peptides and MS/MS were 1.2 and 0.6. The peptide charge was equal to 1^+^, 2^+^, or 3^+^. The altered protein expression (upregulation and downregulation) under different conditions was filtered and analyzed statistically using control-to-treatment comparison (t-test; *p* < 0.05) and one-way ANOVA with the DeCyder MS 2.0 Differential Analysis Software (PepMatch module, GE Healthcare Bio-Science AB). The protein expression levels shown in Table 2, Supplementary Materials Spreadsheets S1 and S2 are the *log2* values. After the significant changes in protein expression were analyzed, the log2-fold change in the expression of each protein in the control and treatment (6 µM 7HF) groups was calculated and treatments that caused more than 1.4-fold change compared to the levels in the control group were selected. All proteins were grouped by their biological functions using the UniProtKB database (http://www.uniprot.org/ (accessed on 30 September 2021)) and the expressed proteins were categorized using a Venn diagram to investigate the functions. PPI networks associated with apoptosis, cell cycle and cell signaling were generated using STRING (Version 11.5, http://string-db.org/ (accessed on 23 October 2021)) and Cytoscape (Version 3.9.0, https://cytoscape.org/ (accessed on 25 October 2021)).

### 4.6. Western Blot Analysis

Protein expression in MDA-MB-468 cells treated with or without 6 µM of 7HF for 0, 12, 24 and 48 h was evaluated. The protein samples were lysates obtained using the radio-immunoprecipitation assay buffer (Thermo Fisher Scientific). The concentrations of the proteins were measured using Bradford assay (Bio-Rad Laboratories, Inc.). Western blotting was performed as described previously by Chimplee et al. (2019) [27]. Briefly, 50 and 100 µg of proteins were separated by 12% SDS-PAGE and blotted onto a nitrocellulose membrane (Bio-Rad). Apart from phosphoproteins used BSA, the membranes were blocked and washed with 5% and 1% low-fat dry milk in 1 × Tween-Tris buffered saline. The proteins were then incubated with various primary antibodies (Cell Signaling Technology, Inc., Danvers, MA, USA). Incubation was performed for 2 h or overnight with antibodies at various concentrations 1:500 (for anti-procaspase-7, 8 and 9 antibodies and antibodies against the cleaved forms), 1:1000 (for anti-Bcl-2, anti-Bax, anti-p53, anti-p21, anti-PP2A-A, anti-FoxO3a, anti-Akt, anti-p-Akt, anti-cyclin B1, anti-Bub3, anti-p-Cdk1 and anti-β actin antibodies) and 1:2000 (for anti-β-catenin antibodies). The proteins were then treated with an anti-IgG rabbit secondary polyclonal antibody (GE Healthcare Ltd., Little Chalfont, Bu, UK; 1:5000) for 1 h. After washing, the protein bands were observed using a Chemiluminescent Detection Kit (Thermo Fisher Scientific). The relative protein expression was analyzed using the Fusion Capt Advanced Quantitation Analysis program (Vilber Lourmat Sté, Collégien, France). Three independent experiments were performed.

### 4.7. Statistical Analysis

All experiments were replicated three or more times. The results of the MTT assay are expressed as the mean ± standard deviation. Data were analyzed by one-way ANOVA using GraphPad Prism 5 (GraphPad Software, San Diego, CA, USA). Differences were considered significant at *p* < 0.05.

## 5. Conclusions

We found that 7HF possessed anti-breast cancer activity. Additionally, 7HF exhibited the strongest cytotoxicity toward TNBC (MDA-MB-468) cells and low cytotoxicity toward normal cells. To the best of our knowledge, this is the first study to report the 7HF activity-molecular mechanism of action network. The molecular mechanism of action of 7HF in MDA-MB-468 cells treated with a low dose (6 µM) seemed to be associated with G2/M-phase arrest (upregulation of Bub3, cyclin B1, p-Cdk1 (Tyr 15) and p53-independent p21 expression), reduction of cell survival (upregulation of PP2A-A and downregulation of p-Akt (Ser 473), FoxO3a and β-catenin expression) and apoptosis (upregulation of Bax and cleaved caspase-7, 8 and 9 expression and downregulation of Bcl-2 and full-length caspase-7, 8 and 9 expression). Proteomic analysis of TNBC cells treated with low-dose 7HF revealed the major protein clusters associated with G1/S-phase arrest and G2/M-phase transition (e.g., mitotic spindle assembly, microtubule dynamics, mitotic chromosome separation and intrinsic and extrinsic apoptosis). We believe that the findings of this study will be useful in subsequent investigations of the effects of 7HF treatment on cancer cells.

## Figures and Tables

**Table 1:** Cytotoxicities and selectivity indices of 7-α-hydroxyfrullanolide (7HF) in human breast cancer and normal cells at 72 h, as evaluated using MTT assay.

Cell Lines	Concentration of 7HF in µg/mL ± SD (µM)			
	IC_20_	IC_50_^a^	IC_90_	SI ^b^
Breast cancer cells				
MCF-7	1.73 ± 0.09 ^c^ (6.97)	4.05 ± 0.13 ^c^ (16.31)	12.58 ± 1.45 ^c^ (50.66)	3.21
MDA-MB-468	1.15 ± 0.24 ^c^ (4.63)	2.97 ± 0.49 ^c^ (11.96)	10.52 ± 1.23 ^c^ (42.36)	4.37
MDA-MB-231	1.63 ± 0.27 ^c^ (6.56)	4.35 ± 0.74 ^c^ (17.52)	16.09 ± 2.92 ^c^ (64.80)	2.99
Normal breast cells				
MCF-12A	4.28 ± 1.20 (17.24)	12.99 ± 7.42 (52.32)	27.15 ± 1.15 (109.33)	ND

Data are presented as the mean ± SD from at least two to three independent experiments. Mean differences were statistically analyzed using one-way ANOVA and Bonferroni’s multiple comparisons test. IC_20_, IC_50_ and IC_90_: 20%, 50% and 90%-maximal inhibitory concentrations, respectively; SI: selective index; ND: not detected ^a^ IC_50_ (µg/mL): <5 = high cytotoxicity; 5–10 = moderate cytotoxicity; >10–25 = weak cytotoxicity [27,33,34]; ^b^ SI: >3 = good selectivity [35,36]; the SI values were calculated by dividing the IC_50_ (in µg/mL) in MCF-12A cells (normal breast cells) by the IC_50_ in breast cancer cells; ^c^ *p* < 0.05, difference in independent IC_20_, IC_50_ and IC_90_ values of cancer cells compared with the corresponding values of the normal cell line (MCF-12A).

**Table 2:** Cell cycle-, apoptosis- and cell signaling-related proteins significantly expressed in 6 µM 7-α-hydroxyfrullanolide-treated cells at 24 h. Fold changes in the expression of differentially expressed proteins compared with the expression in the control. +; upregulated expression, *; the cell signaling proteins were associated with both cell cycle and apoptosis pathways. More cellular signaling proteins were available in Supplementary Materials Spreadsheet S2.

Protein Best Hit	Gene Name	ID Detail Best Hit	Peptide	ID Score	Fold Change
**Cell cycle-related proteins**					
gi|119622929	*CDKN1C*	Cyclin-dependent kinase inhibitor 1C (p57, Kip2)	ASELASR	13.74	+4.0
gi|62087156	*RB1*	Retinoblastoma-associated protein	GVMPPK	15.21	+1.6
gi|21361399	*PPP2R1A*	Serine/threonine-protein phosphatase 2A 65 kDa regulatory subunit A alpha	IGPILDNSTLQSEVKPILEK	17.13	+7.0
gi|530401711	*RAN*	GTP-binding nuclear protein Ran	NLQYYDISAK	17.71	+6.9
gi|578821456	*NUMA1*	Nuclear mitotic apparatus protein 1	GRAQADLALEKAARAELEMR	9.41	+4.7
gi|126116596	*ASPM*	Abnormal spindle-like microcephaly-associated protein	GFIQKR	25.65	+1.5
gi|578824487	*ESPL1*	Separin/Separase	AVRADTGQER	11.63	+3.0
gi|11119736	*NCAPG*	Chromosome condensation protein G	TAALXK	2.57	+1.4
gi|578823215	*CIT*	Citron Rho-interacting kinase	MDQPAK	9.73	+2.6
gi|3378104	*BUB3*	Testis mitotic checkpoint BUB3	VAVEYLDPSPEVQKK	10.09	+8.0
gi|530360485	*MAD2L2*	Mitotic spindle assembly checkpoint protein MAD2B	NMEKIQVIK	24.02	+1.6
gi|530370916	*TUBA4A*	Tubulin alpha-4A chain	LISQIVSSITASLR	72.15	+1.7
gi|124504595	*MAP9*	MAP9 protein	EAKKIAA	10.93	+3.7
gi|530424971	*TUBB6*	Tubulin beta-6 chain	LHFFMPGFAPLTSR	41.79	+3.2
gi|574584816	*TUBB4A*	Tubulin beta-4A chain	AVLVDLEPGTMDSVR	40.58	+1.4
gi|578801711	*ARHGEF2*	Rho guanine nucleotide exchange factor 2	ALVELLREK	12.23	+9.9
gi|392933947	*CTCFL*	Transcriptional repressor CTCFL	KRKQTILK	12.55	+1.4
gi|6912494	*MAPRE1*	Microtubule-associated protein RP/EB family member 1	QGQETAVAPSLVAPALNKPK	25.84	+1.4
gi|578828385	*TELO2*	Telomere length regulation protein TEL2 homolog	QGPAGSPSR	16.00	+1.4
gi|530398458	*TBRG1*	Transforming growth factor beta regulator 1	ENNKLEVLKK	13.93	+1.5
gi|578801489	*CDC73*	Parafibromin	IAAIKAKIMAKK	4.02	+1.5
gi|530411491	*CLTC*	Clathrin heavy chain 1	KFDVNTSAVQVLIEHIGNLDR	43.35	+1.5
gi|23397574	*SNX33*	Sorting nexin-33	ALKGRALYDFHSENK	7.53	+1.6
gi|578829076	*SMPD3*	Sphingomyelin phosphodiesterase 3	GQTPNHNQQDGDSGSLGSPSA′SR	5.18	+2.0
gi|40354195	*KRT18*	Keratin, type I cytoskeletal 18	YALQMEQLNGILLHLESELAQTR	31.75	+2.2
gi|558695404	*CENPP*	Centromere protein P	MVTFQLEFQILEIQNK	6.20	+2.4
gi|68299759	*EVI5*	Ecotropic viral integration site 5 protein homolog	MVTNK	1.99	+3.6
gi|530420984	*FAM9B*	Protein FAM9B	EMKLLRDQFVK	12.68	+7.7
gi|578834213	*HAUS5*	HAUS augmin-like complex subunit 5	KLELEAAVTRLR	10.90	+8.4
**Apoptosis-related proteins**					
gi|90903231	*HTT*	Huntingtin	AVAEPVSR	17.20	+1.8
gi|4506773	*S100A9*	Protein S100-A9	NIETIINTFHQYSVK	66.71	+12.4
gi|530380137	*VDAC1*	Voltage-dependent anion-selective channel protein 1	VTQSNFAVGYK	49.48	+1.4
gi|9956035	*GAPDH*	Glyceraldehyde-3-phosphate dehydrogenase	GALQNIIPASTGAAK	11.17	+1.4
gi|9956035	*GAPDH*	Glyceraldehyde-3-phosphate dehydrogenase	LVINGNPITIFQER	10.82	+1.6
gi|374253794	*BCAP31*	B-cell receptor-associated protein 31	LQAAVDGPMDK	24.40	+1.5
gi|388596700	*CASP7*	Caspase-7	AAPPSAAPR	10.49	+1.4
gi|530371043	*CASP8*	Caspase-8	EVLMNFQMTLDK	5.20	+9.1
gi|530432879	*KRT20*	Keratin, type I cytoskeletal 20	MAMQNLNDR	20.42	+15.9
gi|241982780	*PDCD6IP*	Programmed cell death 6-interacting protein isoform 2	FYNELTEILVR	26.52	+1.4
gi|530406156	*UACA*	Uveal autoantigen with coiled-coil domains and ankyrin repeats	TALMLGCEYGCRDAVEVLIK	1.08	+1.5
gi|50428935	*MAP1S*	Microtubule-associated protein 1S	AESKESVGSRDSSKR	20.35	+1.7
gi|545479138	*CCAR1*	Cell division cycle and apoptosis regulator protein 1	GLKSQLIAR	12.66	+1.9
gi|578819125	*FGFR2*	Fibroblast growth factor receptor 2	DLSDLVSEMEMMKMIGKHK	10.27	+1.9
gi|530373021	*CSRNP1*	Cysteine/serine-rich nuclear protein 1	GGCTLGMALR	14.96	+2.4
gi|578820269	*HIPK3*	Homeodomain-interacting protein kinase 3	GTNEIVAIK	22.31	+4.1
gi|578834177	*CARD8*	Caspase recruitment domain-containing protein 8	WISSL	12.17	+4.2
gi|530406156	*UACA*	Uveal autoantigen with coiled-coil domains and ankyrin repeats	MTLNDTLAKTNR	7.20	+5.5
gi|578839877	*RASSF7*	Ras association domain-containing protein 7	LLGLAAMELK	2.58	+7.5
gi|530388008	*BNIP3L*	BCL2/adenovirus E1B 19 kDa protein-interacting protein 3-like	KSGAMK	11.3	+8.1
**Cell signaling proteins related to cell cycle and apoptosis**					
gi|578833310	*SIRT6*	NAD-dependent protein deacetylase sirtuin-6	LMKHLGLEIPAWDGPR	2.06	+5.6
gi|16033448	*MDM2* *	MDM2 variant FB29	AISETGS	8.51	+1.7
gi|380765197	*YWHAG*	Chain A, crystal structure of a tyrosine 3-monooxygenasetryptophan 5- monooxygenase activation protein, gamma polypeptide	DSTLIXQLLR	37.77	+2.2
gi|578840292	*YWHAE*	14-3-3 protein epsilon	VAGMDVELTVEER	86.17	+1.6
gi|578840292	*YWHAE*	14-3-3 protein epsilon	AAFDDAIAELDTLSEESYK	18.17	+2.1
gi|578840292	*YWHAE*	14-3-3 protein epsilon	LICCDILDVLDK	55.94	+5.3
gi|169646441	*GDI2*	Rab GDP dissociation inhibitor beta	FVSISDLLVPK	20.86	+1.7
gi|578801498	*VASH2*	Vasohibin-2	MKILKPASAHSPTQVR	11.48	+1.5

## Data Availability

Not applicable.

## Supplementary Materials

Supplementary Materials Figure S1: 7HF concentration-response curve of human breast cancer and normal cells; Supplementary Materials Figure S2: 7HF concentration-response curve of MDA-MB-468 cells for 24 h and 72 h; Supplementary Materials Figure S3: Proteomic analysis workflow in this study; Supplementary Materials Figure S4: Protein expressions were evaluated using Western blotting at 6 µM 7HF for 24 h; Supplementary Materials Figure S5: Protein expression in triple-negative breast cancer cells treated with 6 µM 7HF for 0, 12, 24, and 48 h; Supplementary Materials Spreadsheet S1; Supplementary Materials Spreadsheet S2.

## Sample Availability

Samples of the compounds are not available from the authors.
