# Supplementary material for: Anticancer Effects and Molecular Action of 7-α-Hydroxyfrullanolide in G2/M-Phase Arrest and Apoptosis in Triple Negative Breast Cancer Cells
Source: Molecules. 2022 Jan 9;27(2):407. doi: 10.3390/molecules27020407 (PMC8779136; doi:10.3390/molecules27020407)

Anticancer Effects and Molecular Action of 7-α-Hydroxyfrullanolide in G2/M-Phase Arrest and Apoptosis in Triple Negative Breast Cancer Cells

Siriphorn Chimplee[1], Sittiruk Roytrakul[2], Suchada Sukrong[3], Theera Srisawat[4,5], Potchanapond Graidist[1], Kanyanatt Kanokwiroon[1]*

[1]Department of Biomedical Sciences and Biomedical Engineering, Faculty of Medicine, Prince of Songkla University, Hat Yai, Songkhla, 90110, Thailand

[2]Functional Ingredients and Food Innovation Research Group, National Center for Genetic Engineering and Biotechnology (BIOTEC), National Science and Technology Development Agency (NSTDA), Pathum Thani, 12120, Thailand

[3]Research Unit of DNA Barcoding of Thai Medicinal Plants, Department of Pharmacognosy and Pharmaceutical Botany, Faculty of Pharmaceutical Sciences, Chulalongkorn University, Bangkok, 10330, Thailand

[4]Faculty of Science and Industrial Technology, Prince of Songkla University, Surat Thani Campus, Surat Thani, 84000, Thailand

[5]Faculty of Innovative Agriculture and Fisheries, Prince of Songkla University, Surat Thani Campus, Surat Thani, 84000, Thailand

*Correspondence and requests for materials should be addressed to K.K.

(Email: kanyanatt.k@psu.ac.th)

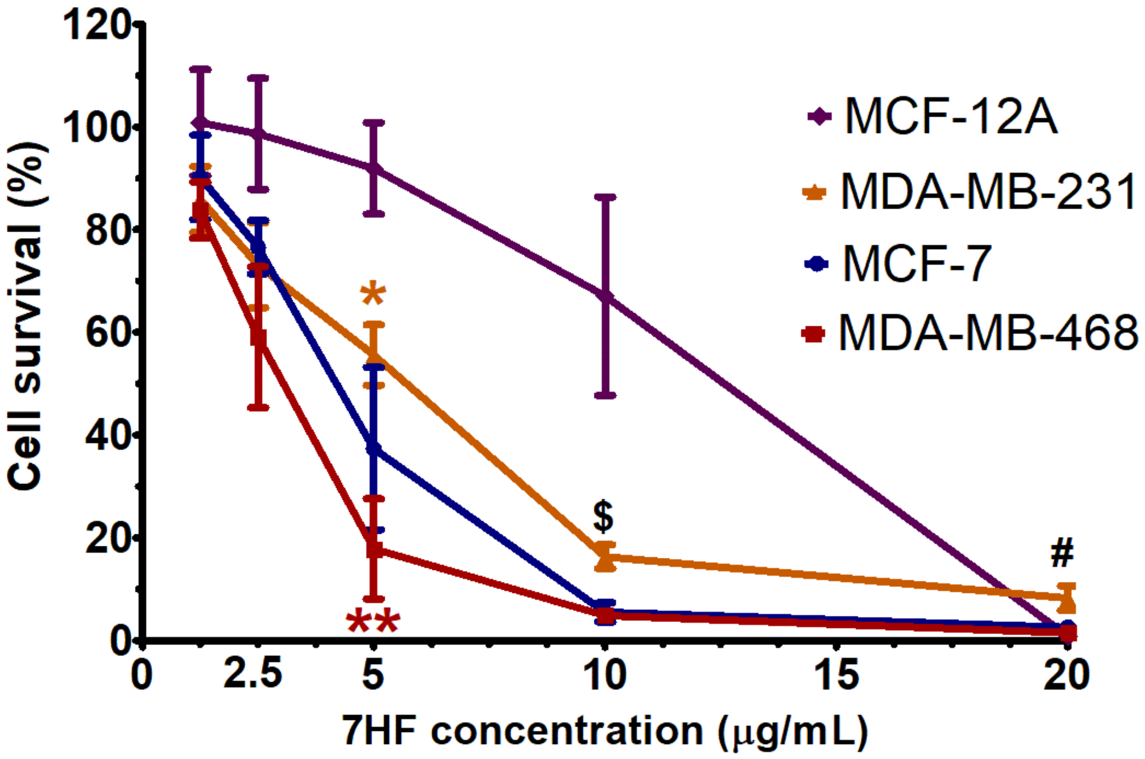

Supplementary Materials Figure S1. 7HF concentration-response curve of human breast cancer and normal cells

The cytotoxicity of 7HF on human breast cancer cells (MCF-7, MDA-MB-468, and MDA-MB-231) and normal cells (MCF-12A) at 72 h. Mean values of cell survivals were derived from two-three independent experiments. Differences were statistically analysed using one-way ANOVA and Dunnett's multiple comparison test. *p<0.05 compared to untreated, **p<0.01 compared to untreated, \$$p$<0.01 7HF (at 10 μg/mL) of all breast cancer cell lines compared to their untreated, and #p<0.01 7HF (at 20 μg/mL) of all cell lines compared to their untreated.

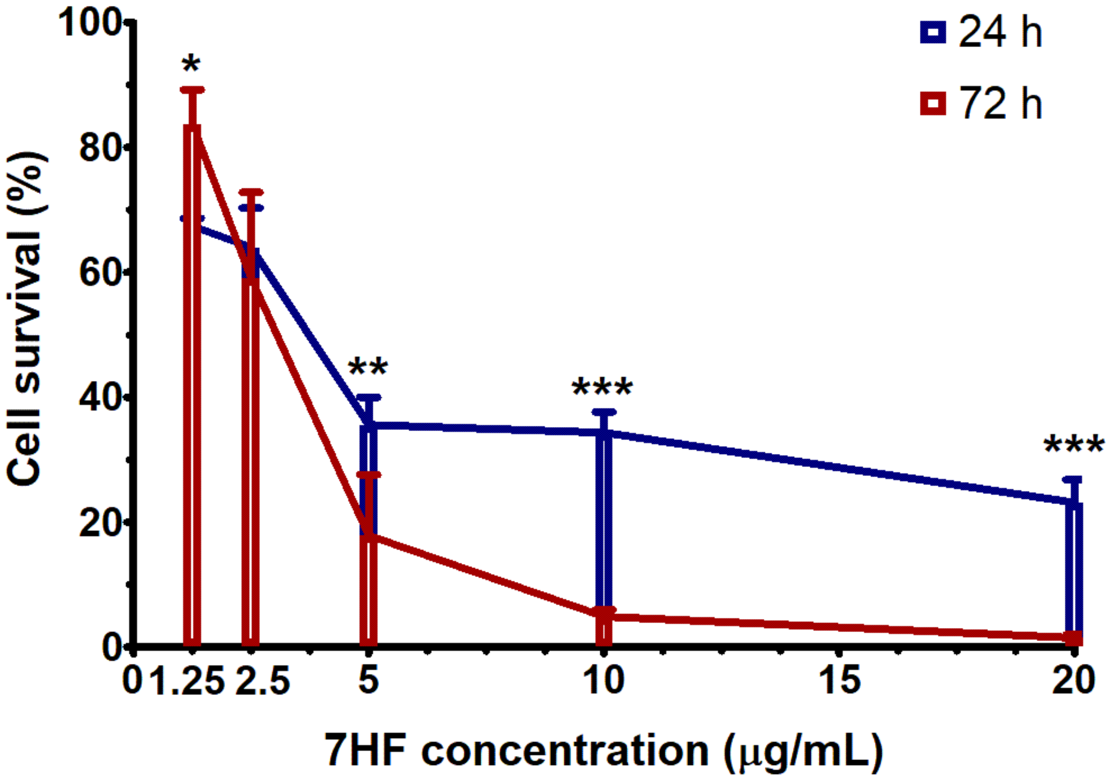

Supplementary Materials Figure S2. 7HF concentration-response curve of MDA-MB-468 cells for 24 h and 72 h. Differences were statistically analysed using two-way ANOVA and Bonferroni posttests comparisons. *, p<0.05, **p<0.01, and *** p<0.001 at 24 h compared to at 72 h.

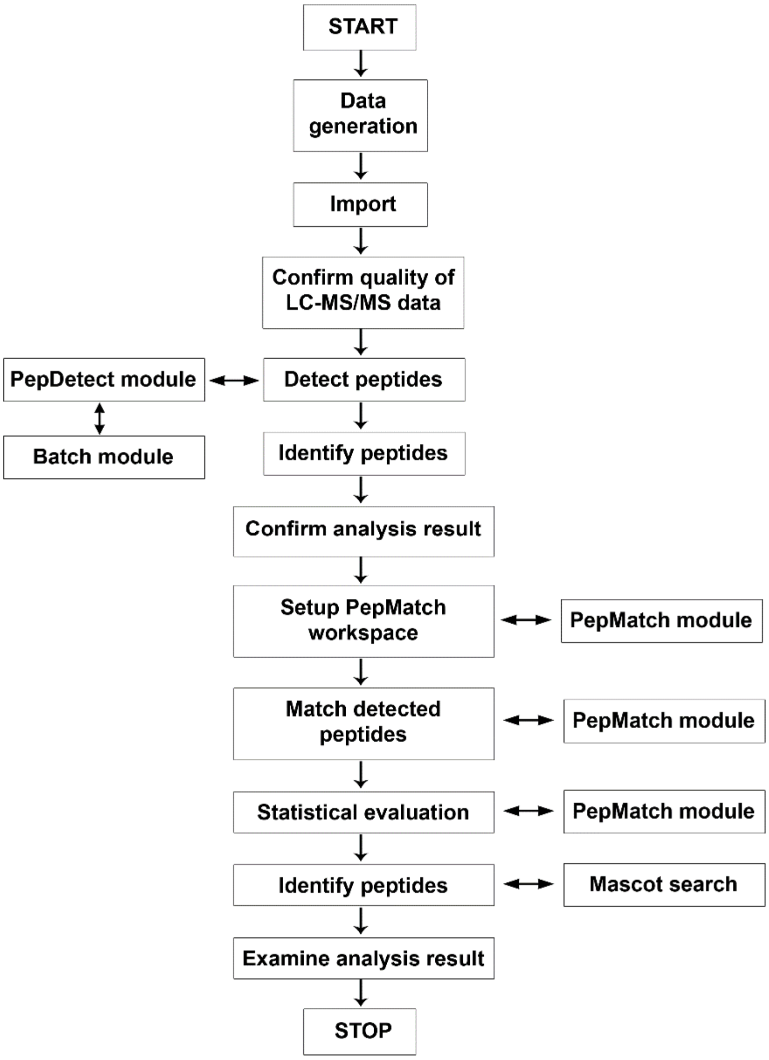

Supplementary Materials Figure S3. Proteomic analysis workflow in this study

DeCyder MS 2.0 Differential Analysis Software and tools (PepDetect, Batch, and PepMatch) were used for proteome identification.

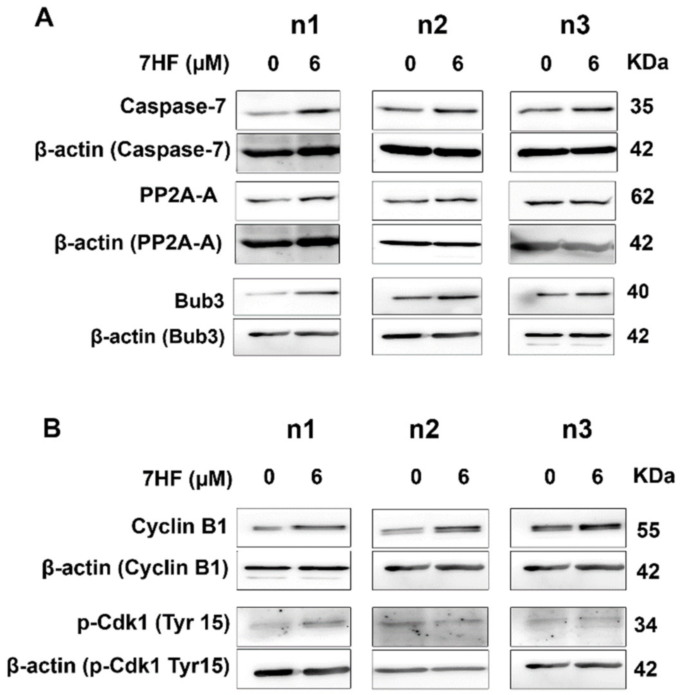

Supplementary Materials Figure S4. Protein expressions were evaluated using Western blotting at 6 μM 7HF for 24 h. (A) PP2A-A, Bub3, and caspase-7 expression was validated using proteomics. **(B)** Cyclin B1 and phosphorylated Cdk1 (p-Cdk1) (Tyr15) expression after 7 HF treatment. These proteins were depicted along with their β-actin. The results were performed in three independent experiments (n1- n3) . The experiment n1-n3 used for calculation of band intensity (or relative expression) from indicated protein and β-actin in Figure 4C-D (page 10). The full original Western blot images show in page 3-7 in this revision as follows: caspase-7, PP2A-A, Bub3, Cyclin B1, and p-Cdk1 (Tyr15). In full original blot, M; Protein marker, 0, 6 μM; 7HF treatment does (0 and 6 μM) at 24 h, Black outer square; border of full images, Red inner square and black head arrow; indicated protein bands; #The blots are shown the same in Figure 4C-D (Page 10).

Caspase 7 (35 KDa) and β-actin (42 KDa)

Caspase 7 (n1)

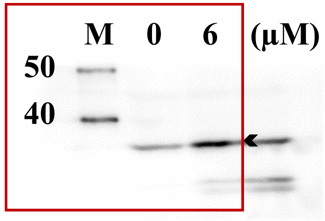

# The result represented in Figure 4C

Caspase 7 (n2)

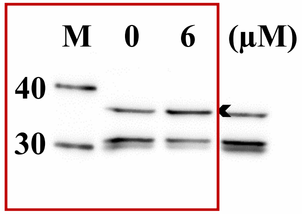

Caspase 7 (n3)

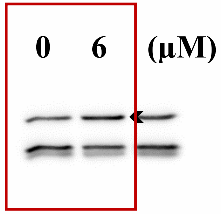

β-actin (n1)

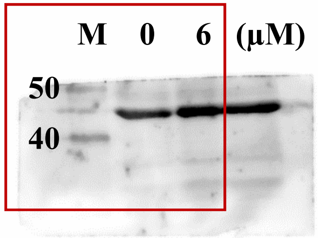

# The result represented in Figure 4C

β-actin (n2)

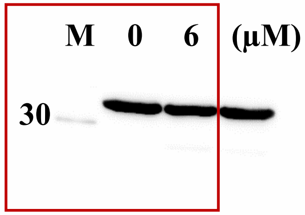

β-actin (n3)

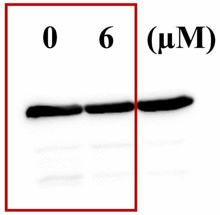

PP2A (62 KDa) and β-actin (42 KDa)

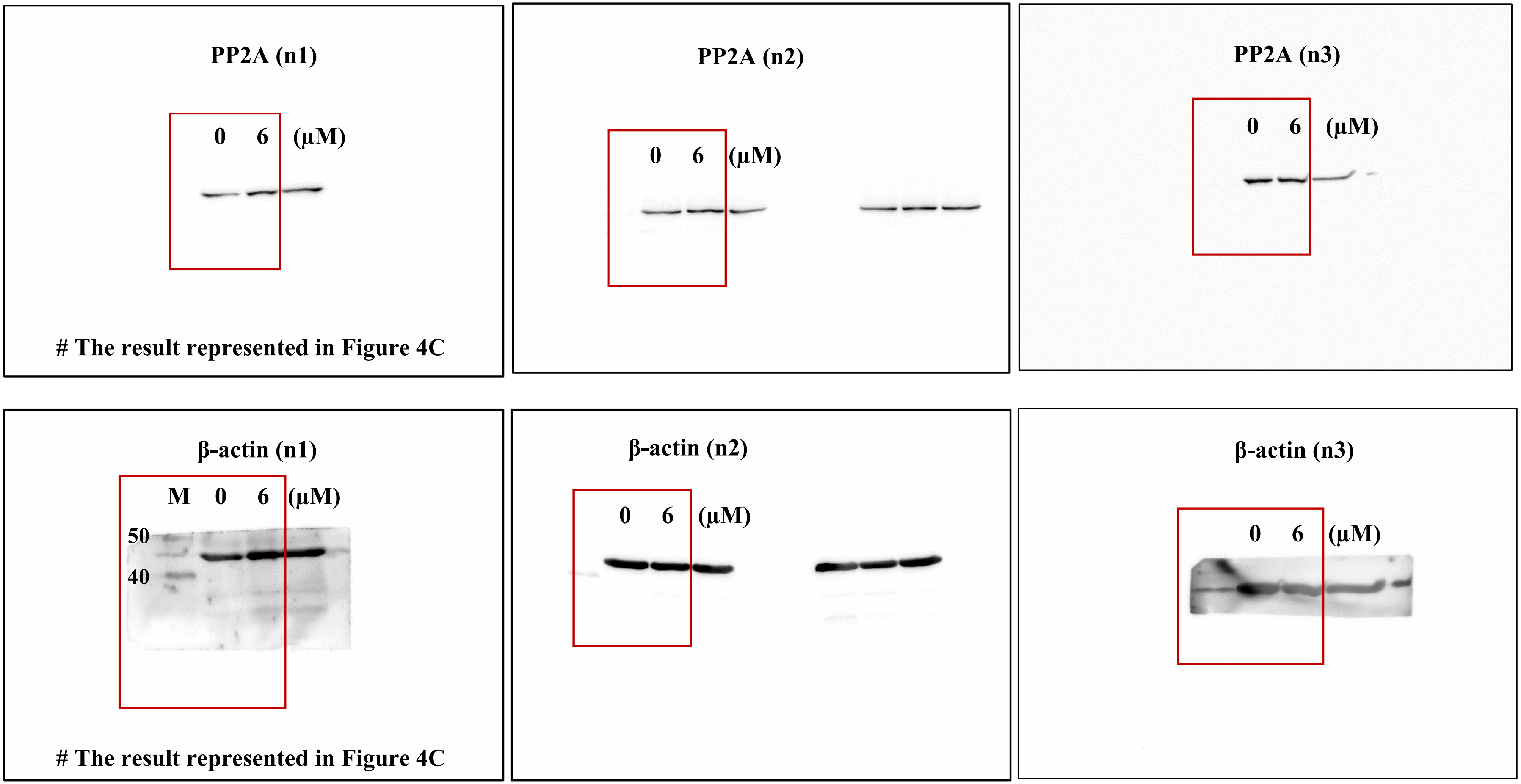

Bub3 (40 KDa) and β-actin (42 KDa)

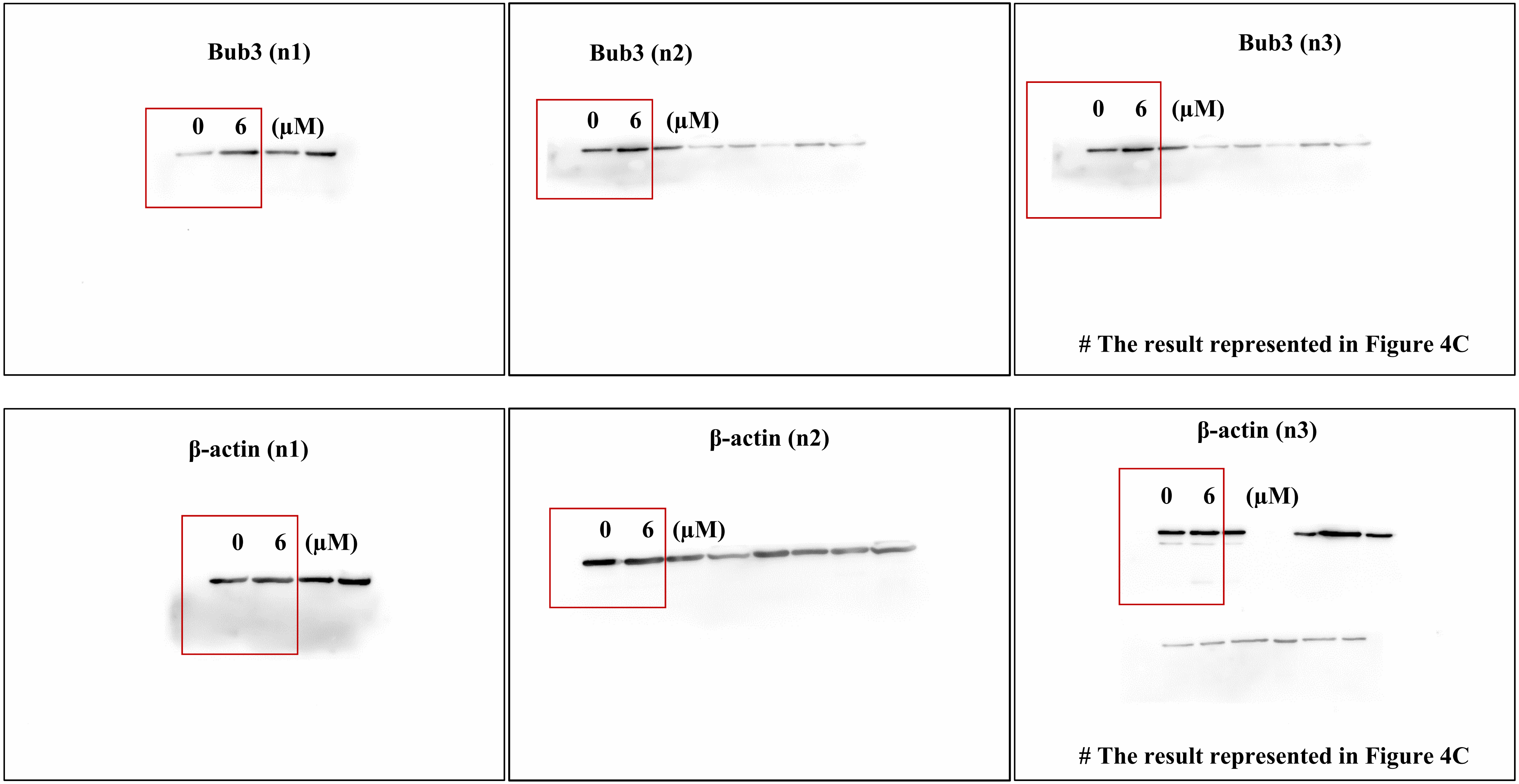

Cyclin B1 (55 KDa) and β-actin (42 KDa)

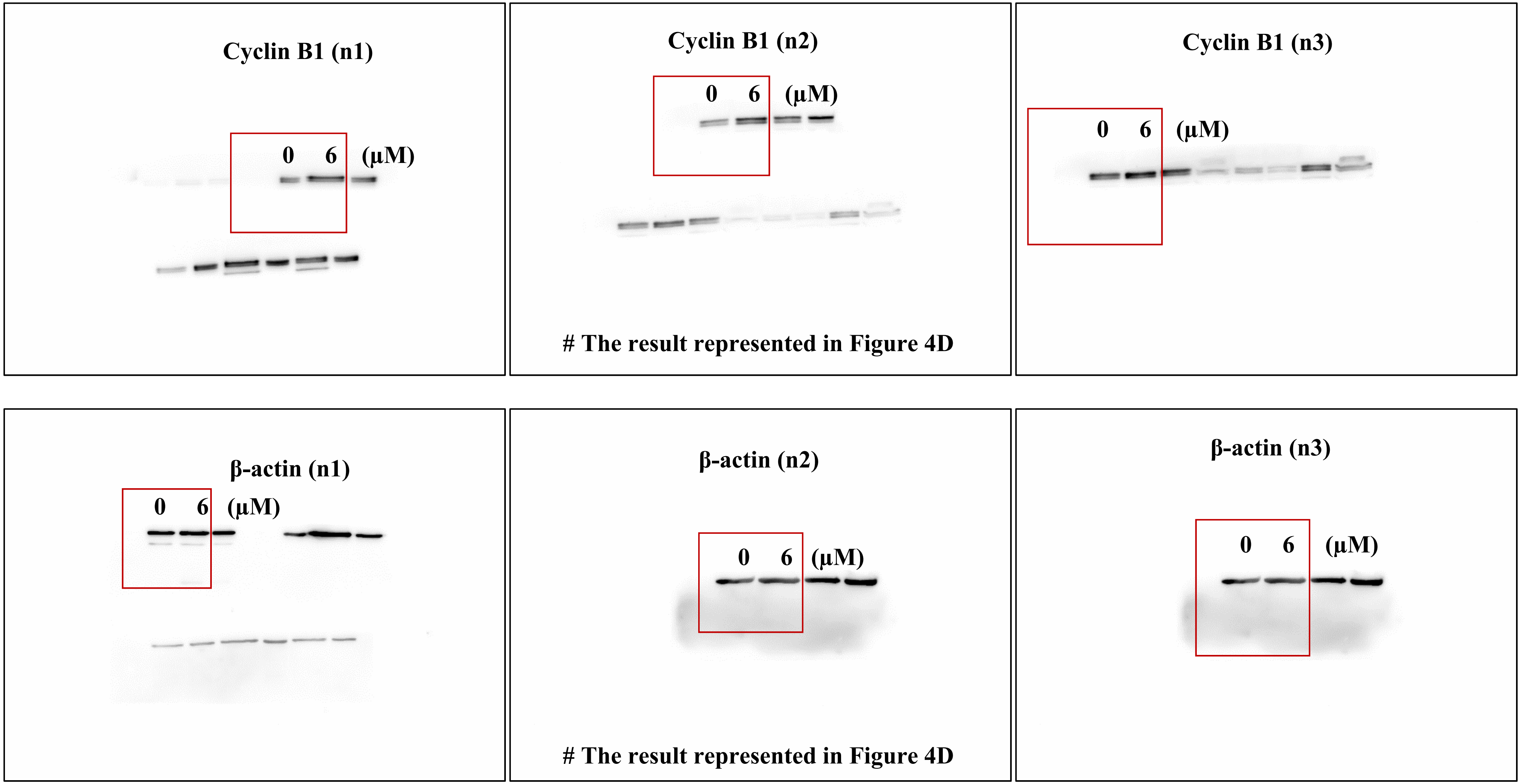

p-Cdk1 (Tyr15) (34 KDa) and β-actin (42 KDa)

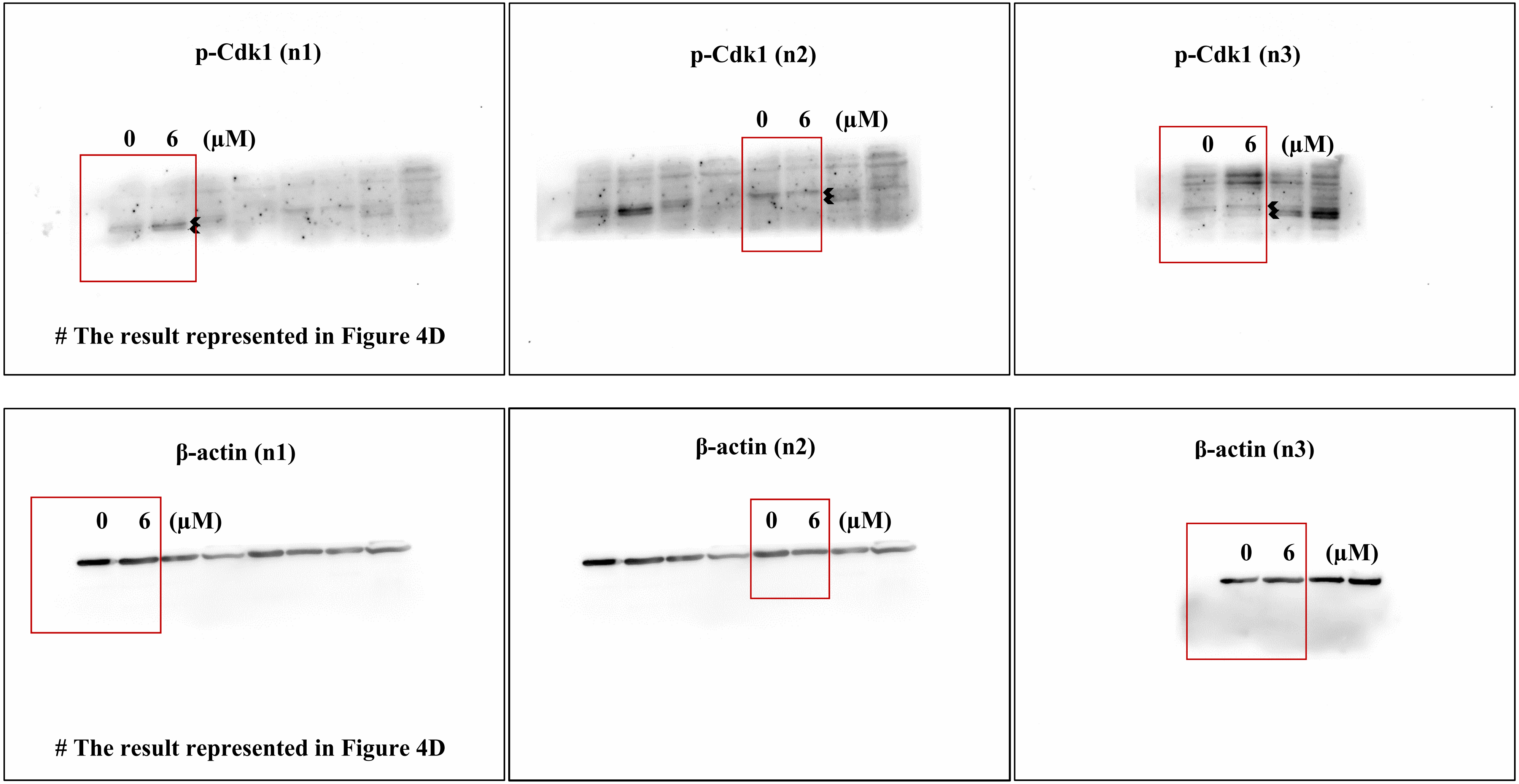

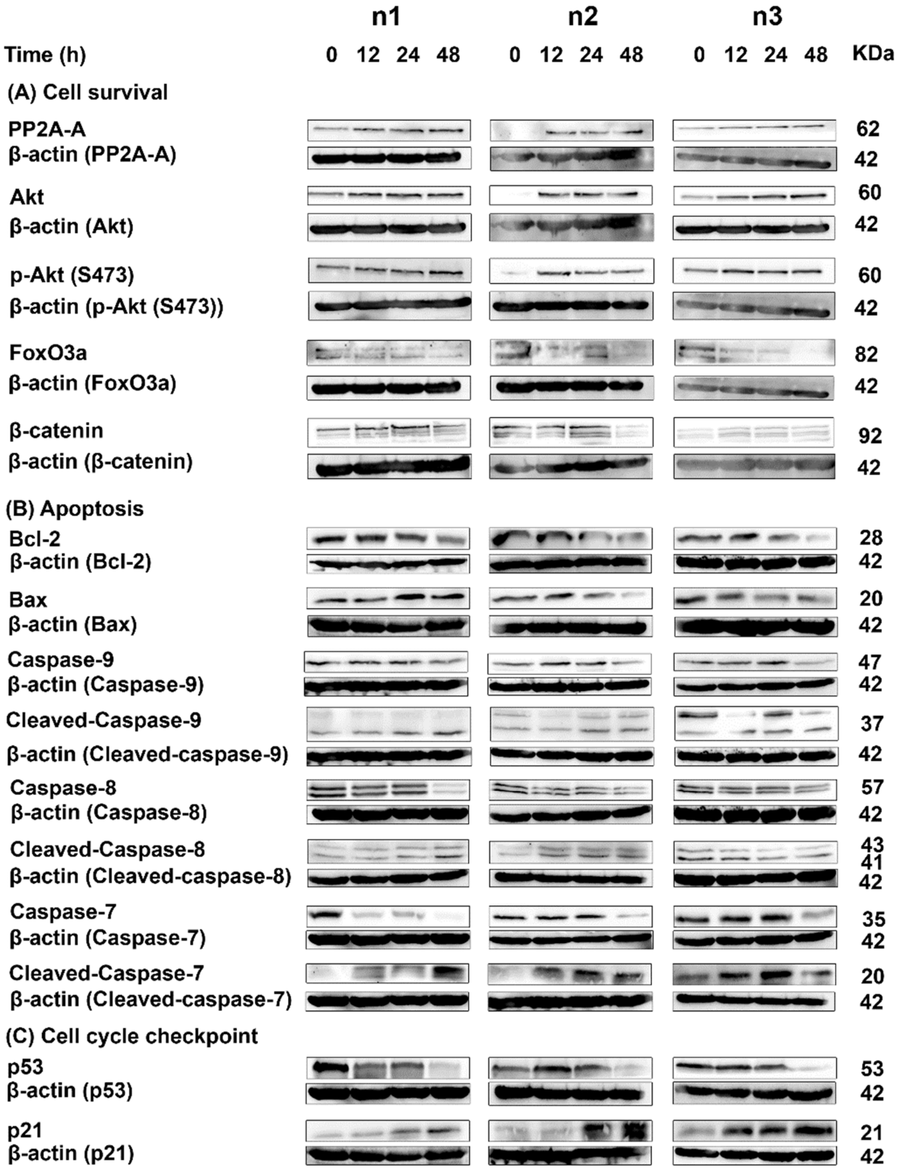

Supplementary Materials Figure S5. Protein expression in triple-negative breast cancer cells treated with 6 µM 7HF for 0, 12, 24, and 48 h. (A) Cell survival-related, **(B)** cell apoptotic, **(C)** cell cycle checkpoint protein expression after 7HF treatment. These proteins were depicted along with their β-actin. The results were performed in three independent experiments (n1-n3). The experiment n1-n3 used for calculation of band intensity (or relative expression) from indicated protein and β-actin in Figure 5A-C (page 11). The full original Western blot images show in page 10-24 in this revision as follows: PP2A-A, Akt, p-Akt (Ser473), FoxO3a, and β-catenin, Bcl-2, Bax, Caspase-9, Cleaved caspase-9, Caspase-8, Cleaved-caspase-8, Caspase 7, Cleaved caspase 7, p53, and p21. In full original blot, M;

Protein marker, 0, 12, 24, 48 h; 7HF treatment times (0, 12, 24 and 48 h) at 6 μM, Black outer square; border of full images, Red inner square and black head arrow; indicated protein bands; #The blots are shown the same in Figure 5A-C (Page 11).

PP2A (62 KDa) and β-actin (42 KDa)

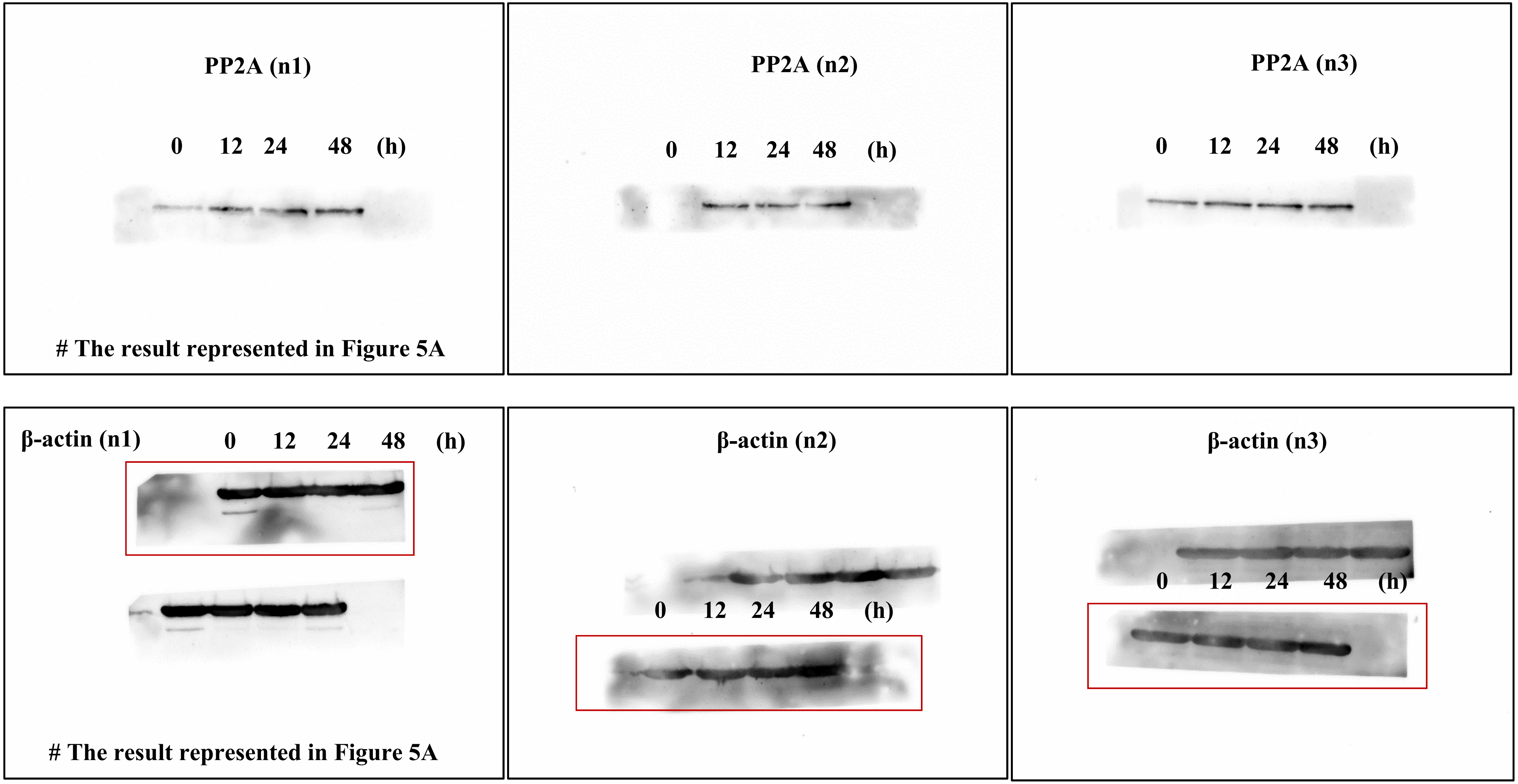

Akt (60 KDa) and β-actin (42 KDa)

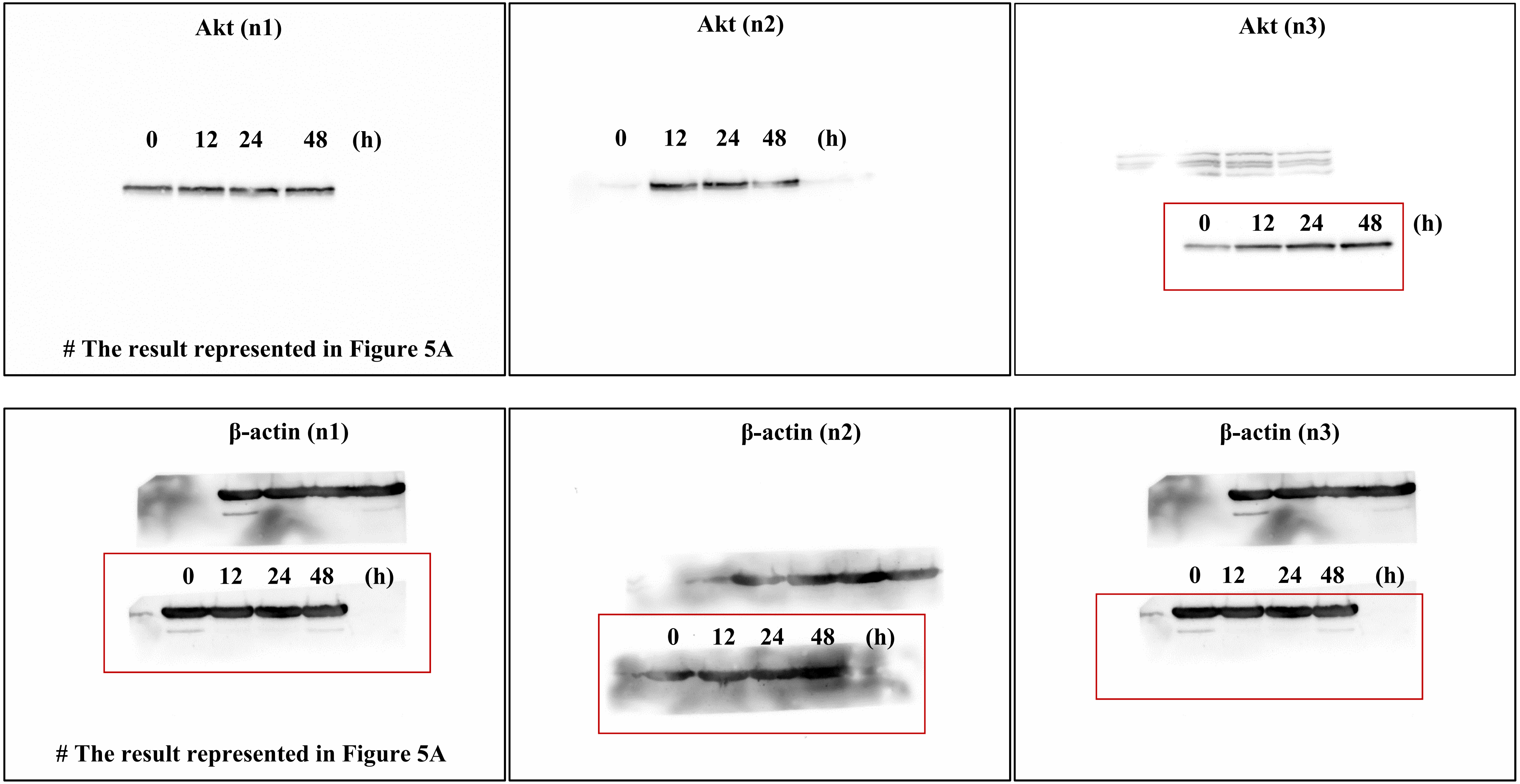

p-Akt (Ser473) (60 KDa) and β-actin (42 KDa)

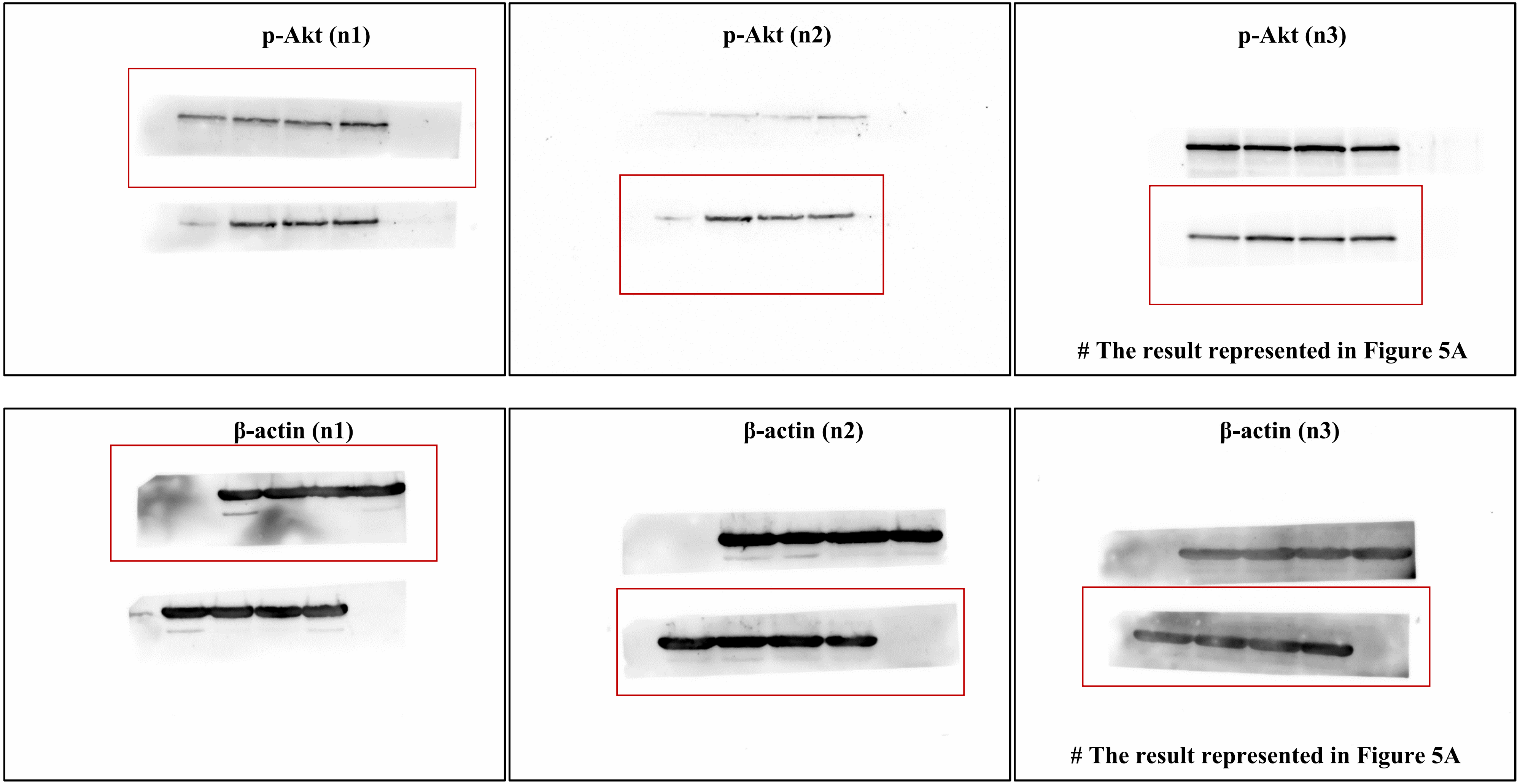

FoxO3a (82 KDa) and β-actin (42 KDa)

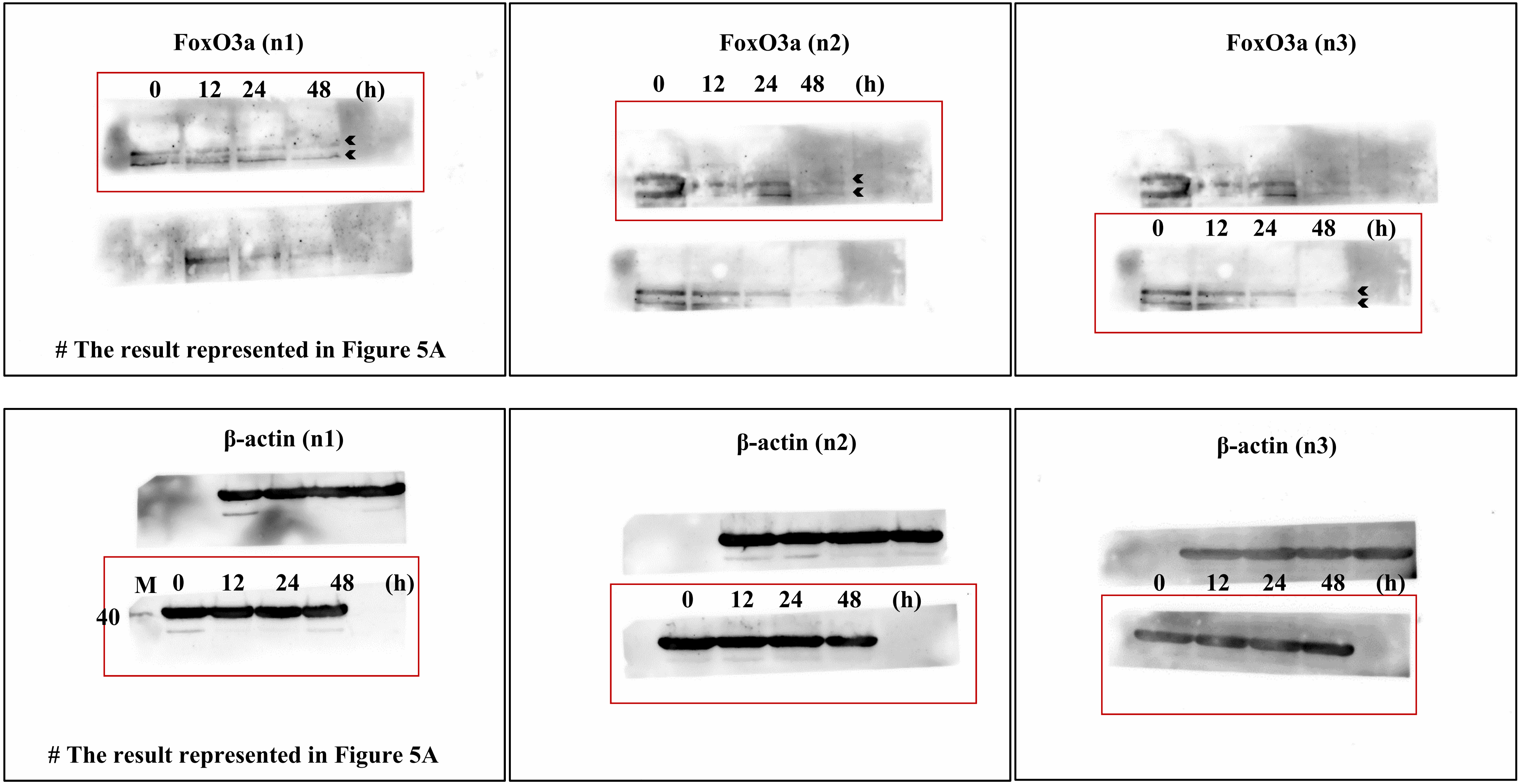

β-catenin (92 KDa) and β-actin (42 KDa)

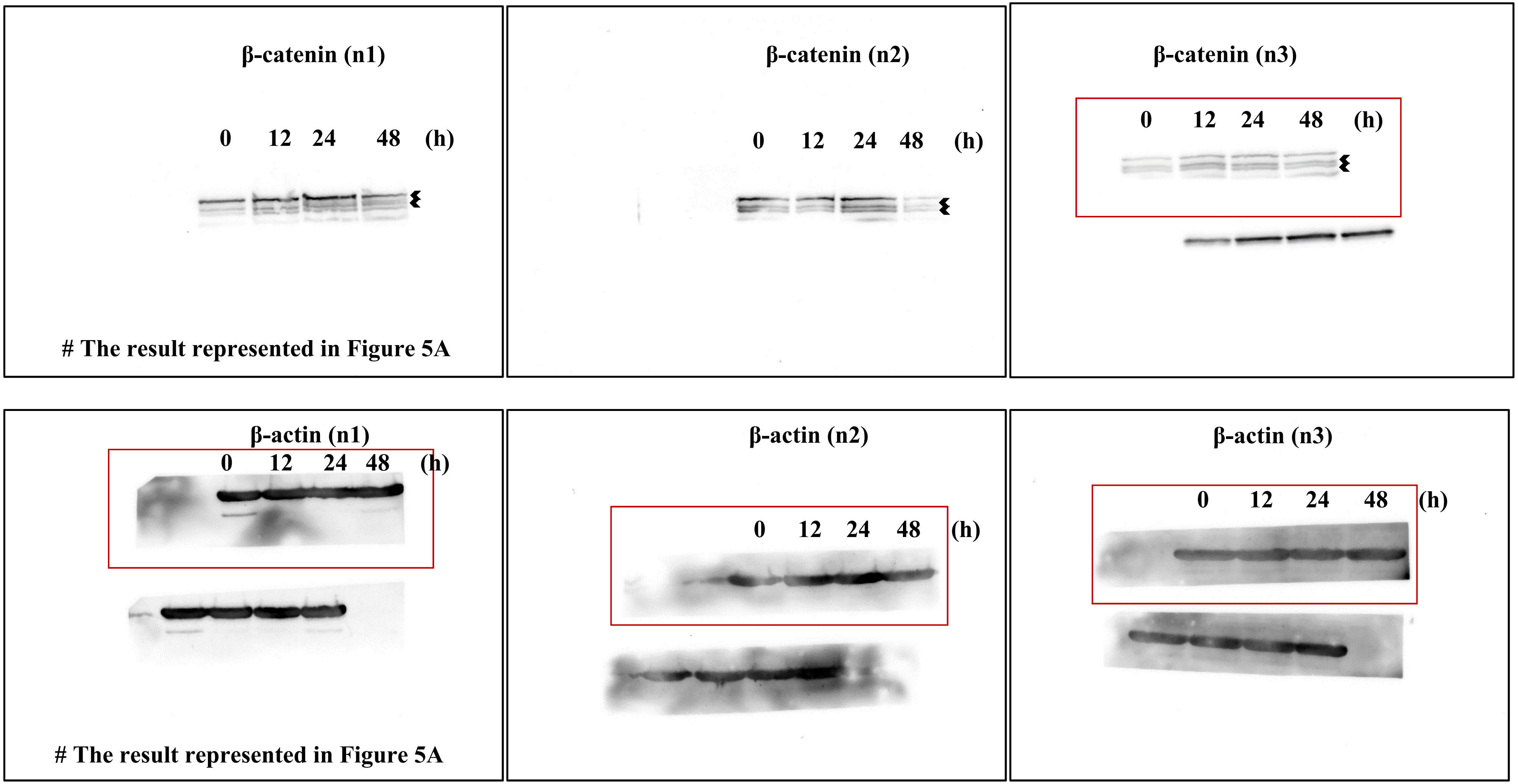

Bcl-2 (28 KDa) and β-actin (42 KDa)

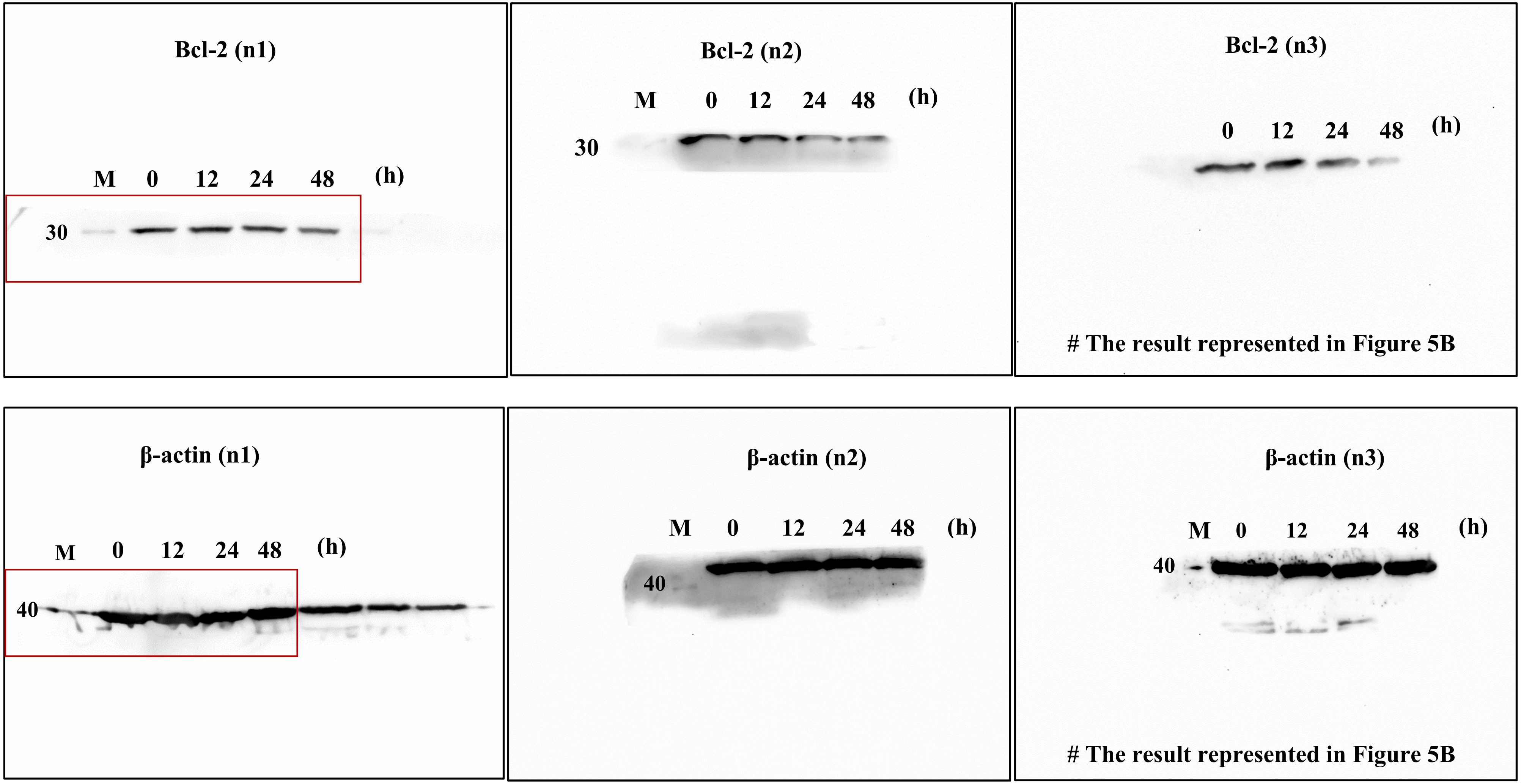

Bax (20 KDa) and β-actin (42 KDa)

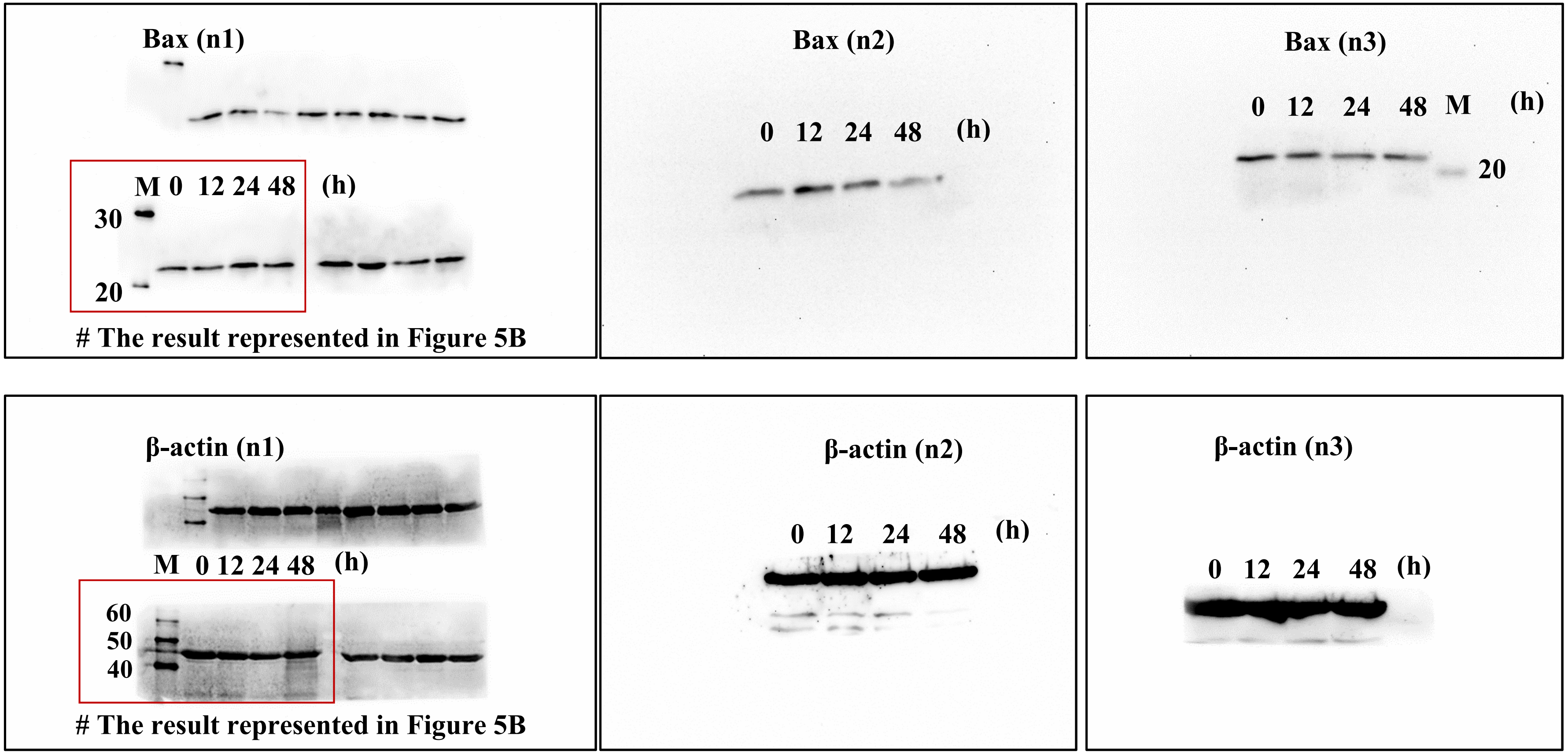

Caspase 9 (47 KDa) and β-actin (42 KDa)

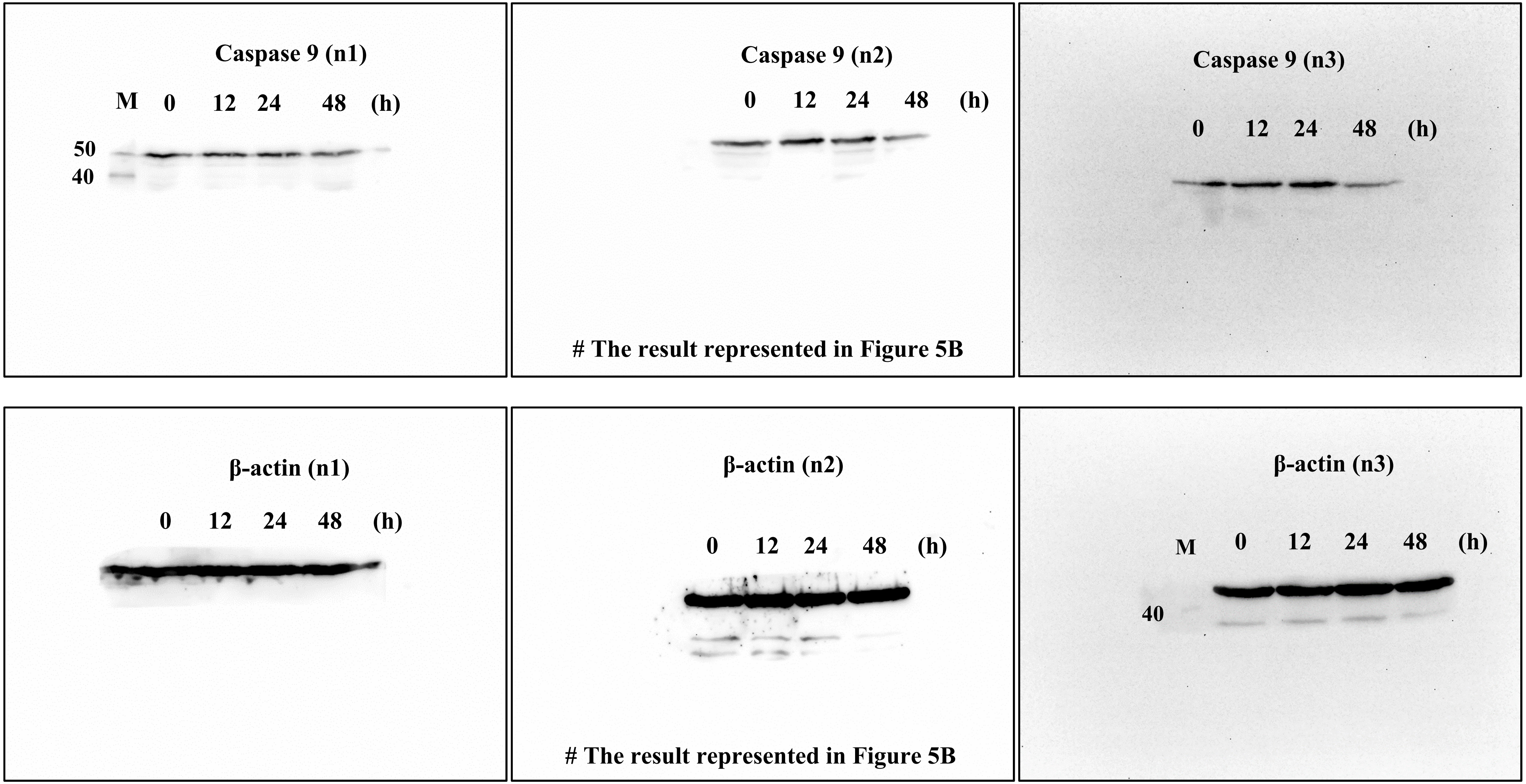

Cleaved caspase 9 (37 KDa) and β-actin (42 KDa)

Cleaved caspase 9 (n1)

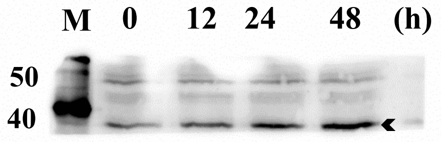

The result represented in Figure 5B

Cleaved caspase 9 (n2)

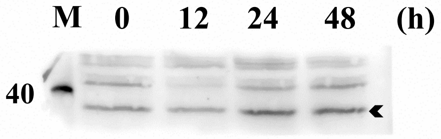

Cleaved caspase 9 (n3)

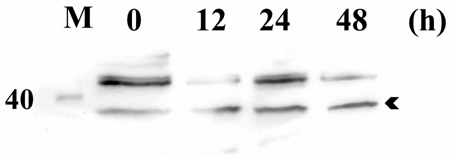

β-actin (n1)

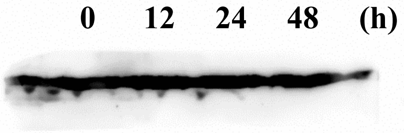

The result represented in Figure 5B

β-actin (n2)

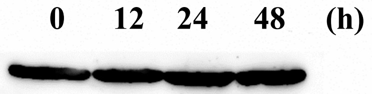

β-actin (n3)

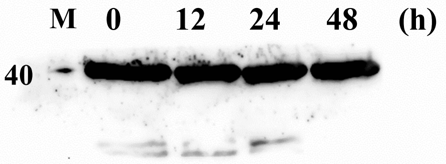

Caspase 8 (57 KDa) and β-actin (42 KDa)

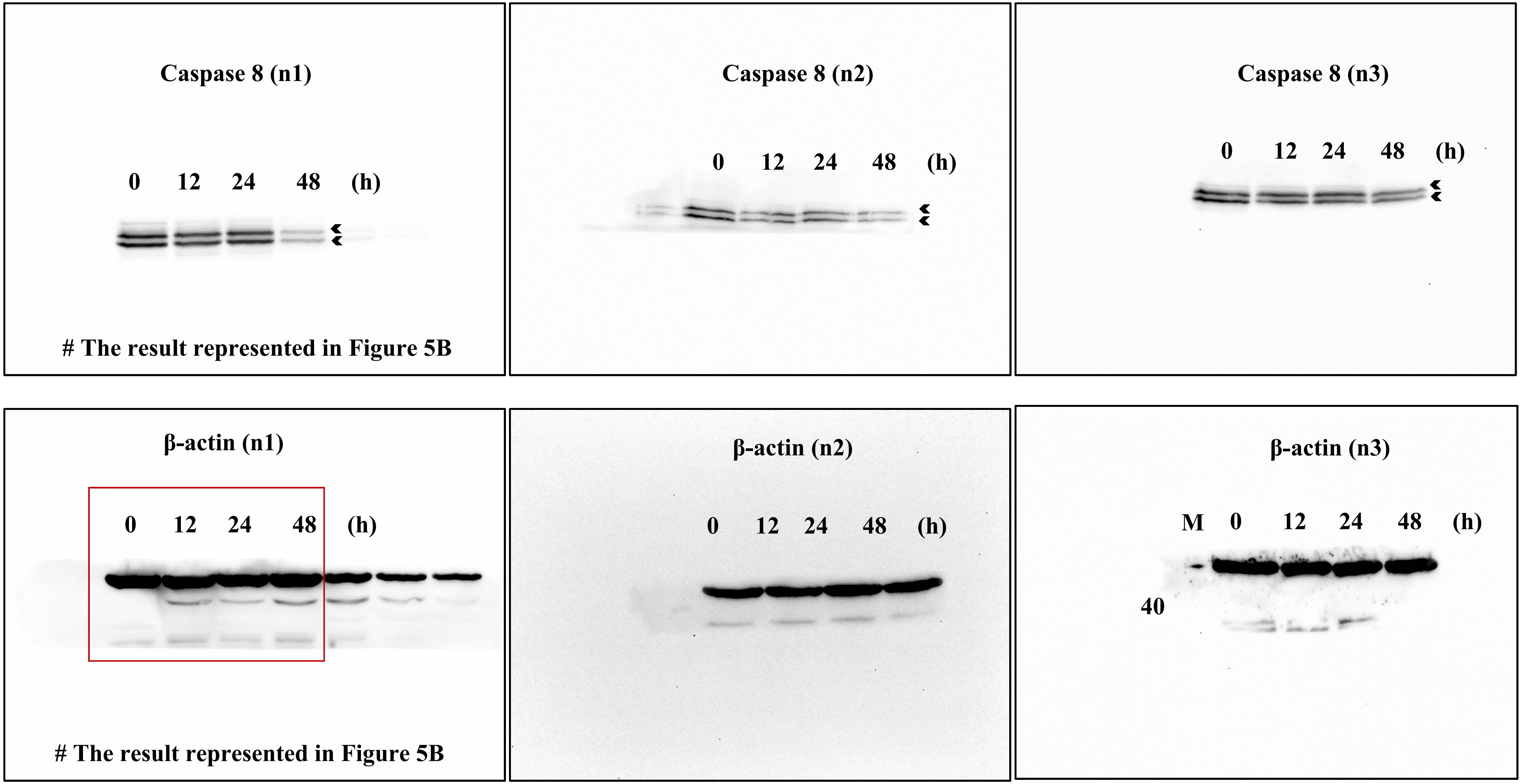

Cleaved caspase 8 (41 and 43 KDa) and β-actin (42 KDa)

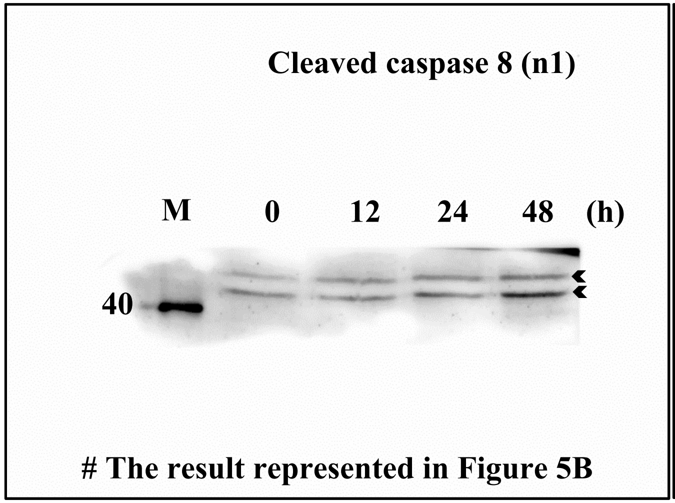

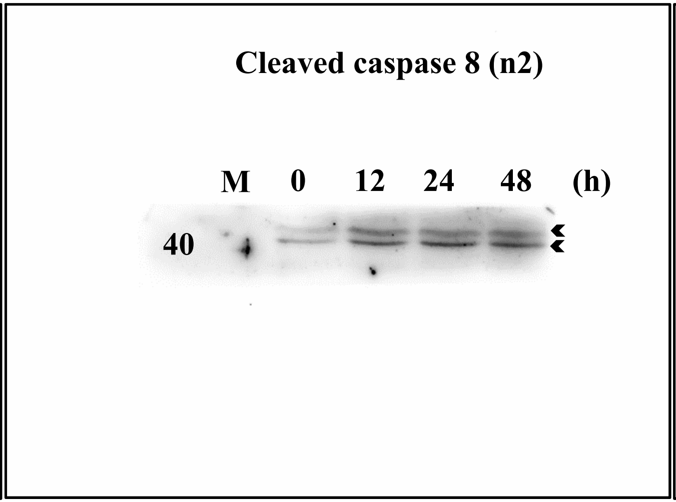

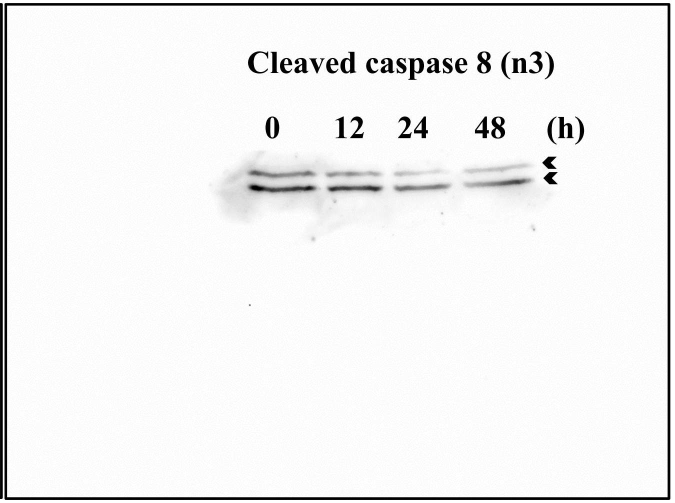

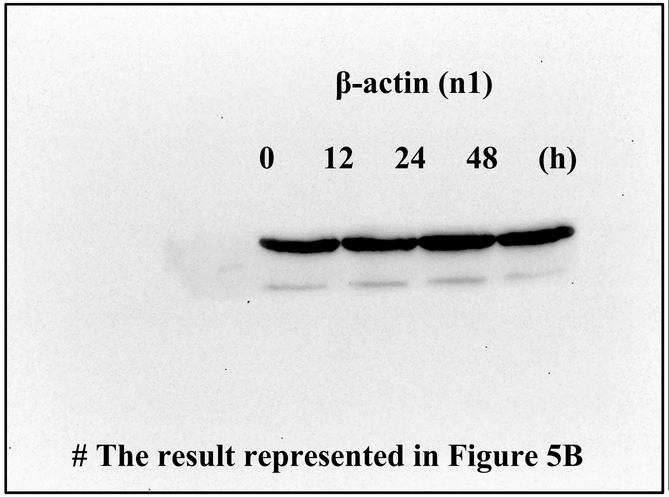

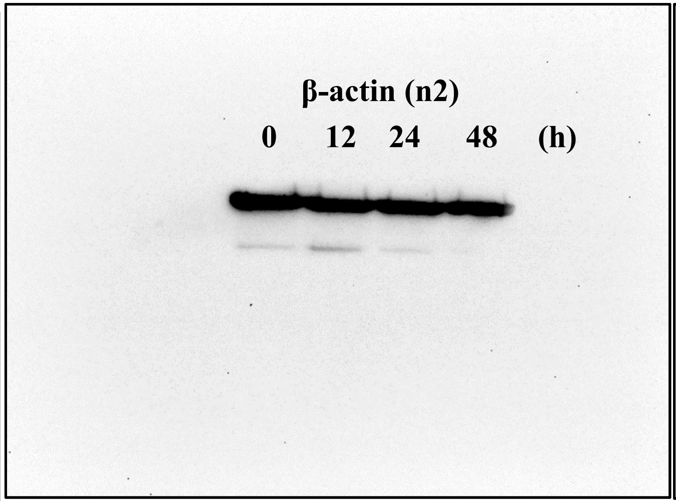

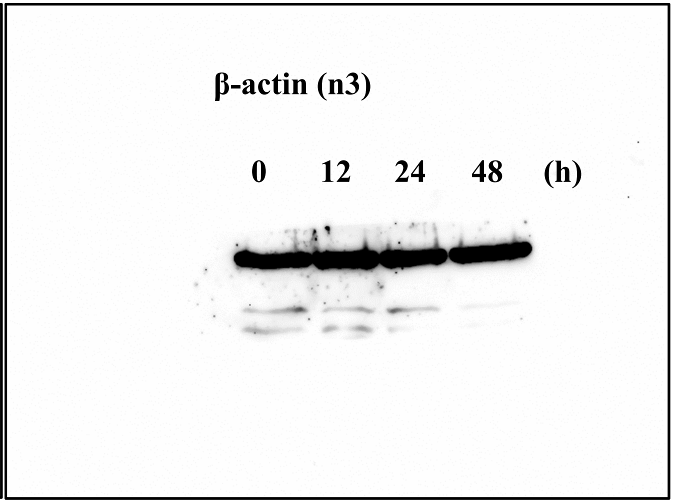

Caspase 7 (35 KDa) and β-actin (42 KDa)

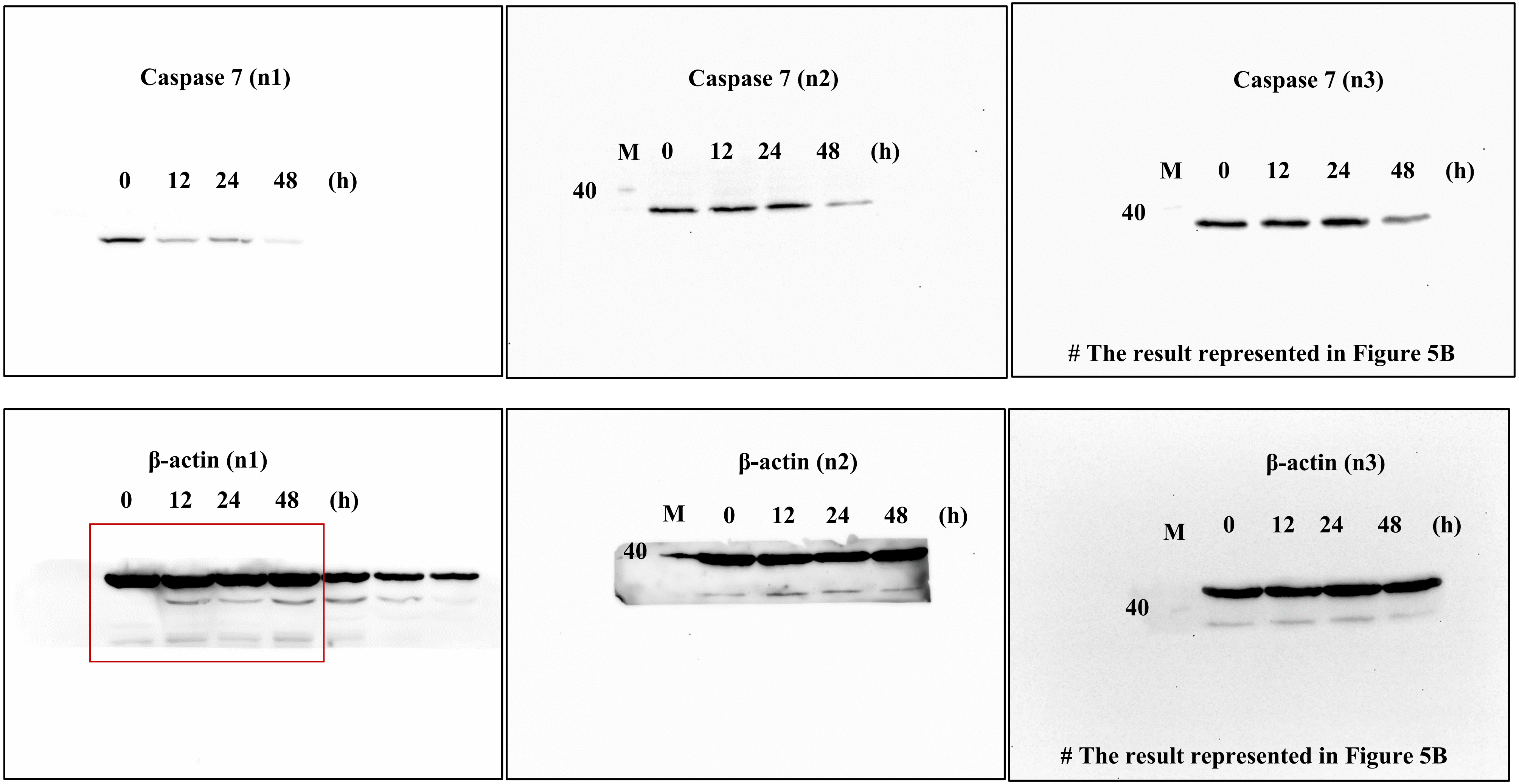

Cleaved caspase 7 (20 KDa) and β-actin (42 KDa)

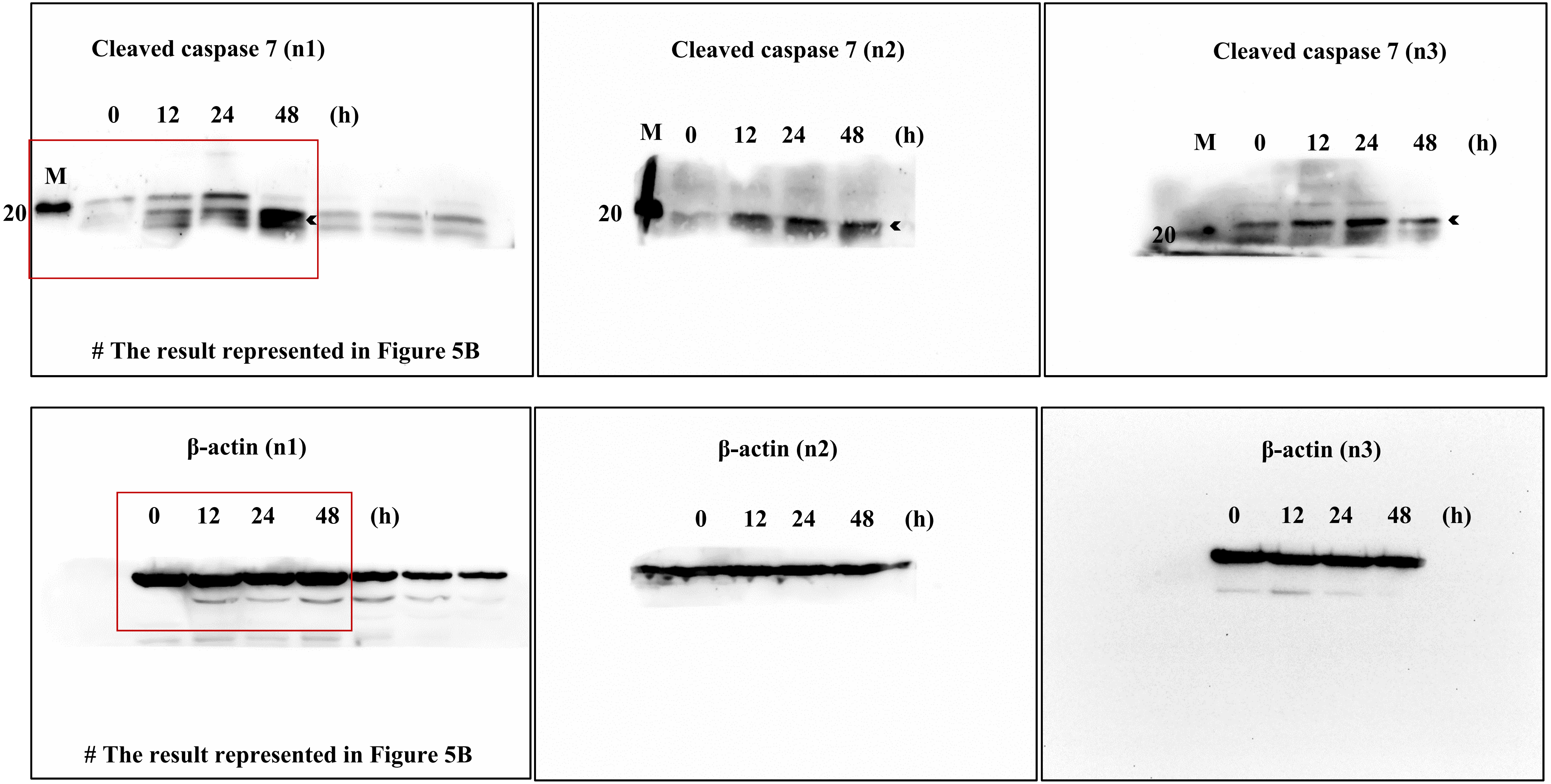

p53 (53 KDa) and β-actin (42 KDa)

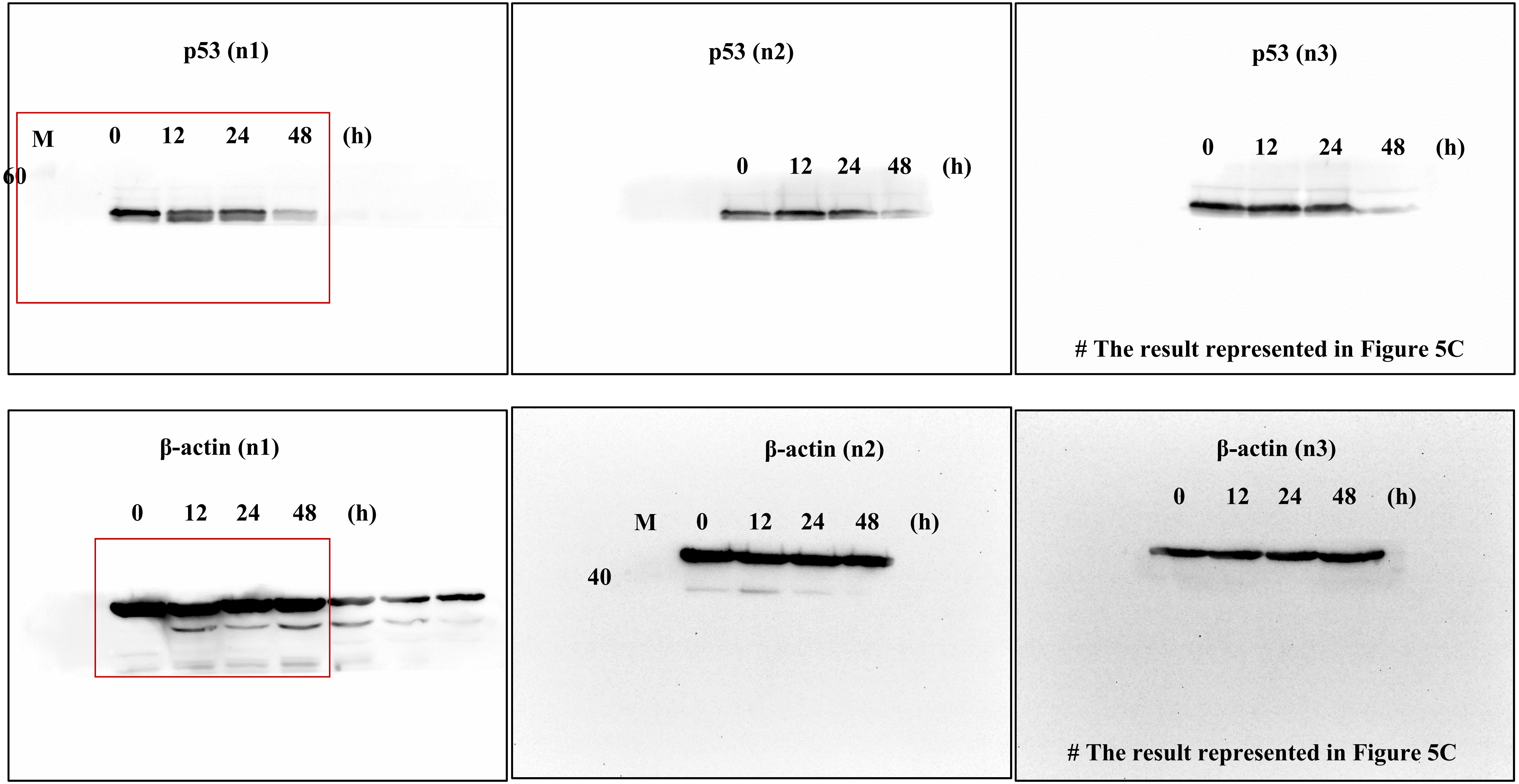

p21 (21 KDa) and β-actin (42 KDa)

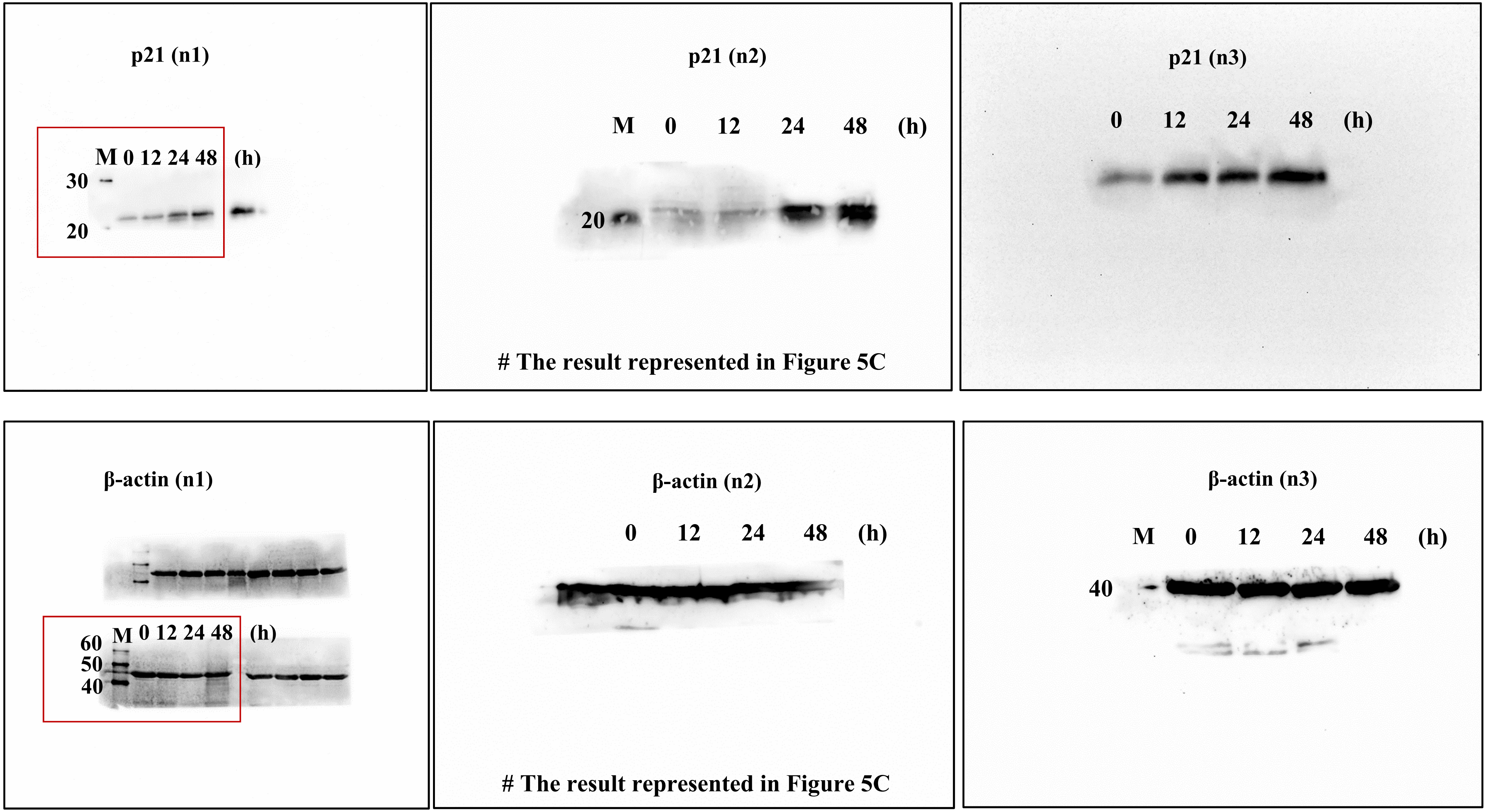

Supplement: Supplementary file 1 [file molecules-27-00407-s001.zip › Supplementary Materials.pdf]
